# Understanding Self‐Assembly of Silica‐Precipitating Peptides to Control Silica Particle Morphology

**DOI:** 10.1002/adma.202207586

**Published:** 2023-01-25

**Authors:** Johannes Strobl, Fanny Kozak, Meder Kamalov, Daniela Reichinger, Dennis Kurzbach, Christian FW Becker

**Affiliations:** ^1^ Institute of Biological Chemistry Faculty of Chemistry University of Vienna Währinger Str. 38 Vienna 109 Austria; ^2^ Vienna Doctoral School in Chemistry (DoSChem) University of Vienna Währinger Str. 42 Vienna 1090 Austria

**Keywords:** biomineralization, molecular dynamics, peptide self‐assembly, silica particles, silica precipitation

## Abstract

The most advanced materials are those found in nature. These evolutionary optimized substances provide highest efficiencies, e.g., in harvesting solar energy or providing extreme stability, and are intrinsically biocompatible. However, the mimicry of biological materials is limited to a few successful applications since there is still a lack of the tools to recreate natural materials. Herein, such means are provided based on a peptide library derived from the silaffin protein R5 that enables rational biomimetic materials design. It is now evident that biomaterials do not form via mechanisms observed in vitro. Instead, the material's function and morphology are predetermined by precursors that self‐assemble in solution, often from a combination of protein and salts. These assemblies act as templates for biomaterials. The RRIL peptides used here are a small part of the silica‐precipitation machinery in diatoms. By connecting RRIL motifs via varying central bi‐ or trifunctional residues, a library of stereoisomers is generated, which allows characterization of different template structures in the presence of phosphate ions by combining residue‐resolved real‐time NMR spectroscopy and molecular dynamics (MD) simulations. Understanding these templates in atomistic detail, the morphology of silica particles is controlled via manipulation of the template precursors.

## Graphical abstract

Biomimetic silaffin‐based peptides give rise to different template structures depending on stereochemistry and connectivity of the central residue. NMR experiments help to decipher the peptidic template structures. The resulting structure–activity relationship provides a key to rationally control the assembly of such peptides and thereby the resulting silica material as the peptidic precursor structures determine the materials final architecture.

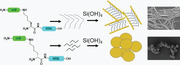

## 1 Introduction

One of the key prerequisites for the existence of biological organisms is the process of controlled self‐assembly,^[^ ^1^, ^2^ ^]^ which enables compartmentalization and, thus, the separation of cellular entities. The capacity of biomolecules to self‐assemble into nanostructures also provides the basis for the in vitro production of (bio‐)nanomaterials. Materials produced in this manner possess high biocompatibility and promise applications in drug delivery, tissue engineering, bioimaging, and biosensors.^[^ ^2^, ^3^, ^4^ ^]^ In this context, small proteins and peptides have recently received ample attention as building blocks for bionanomaterials due to their ability to form highly diverse molecular structures^[^ ^5^, ^6^ ^]^ with tailored properties. Genetic engineering has proven effective too, in designing polypeptides binding inorganic moieties to access tailored nanomaterials with distinct properties.^[^ ^7^, ^8^, ^9^ ^]^

In almost all life forms, the interaction between self‐assembled proteins or peptides and inorganic molecules enables the formation of important hybrid materials that constitute, among others, bones, teeth, silica skeletons, and shells.^[^ ^10^ ^]^ Adaptions of bioshell formation were recently reported, capitalizing on these evolutionary optimized processes to generate highly defined silica nanostructures under mild aqueous conditions in vitro.^[^ ^10^, ^11^ ^]^ One successful example is based on the silaffin R5 peptide derived from the diatom *Cylindrotheca fusiformis* that precipitates defined solid silica phases.^[^ ^12^ ^]^ The R5 peptide is part of the silica‐precipitation machinery of the diatom cell wall,^[^ ^13^ ^]^ and the related in vivo properties could be transferred to the laboratory to produce homogeneous silica particles under mild, biocompatible conditions. This process starkly contrasts traditional methods of industrial silica production that require harsh temperature and pH conditions.^[^ ^14^ ^]^ The silica‐precipitating properties of the R5 peptide have been applied to other materials, too, including titanium salts forming spherical microparticles.^[^ ^15^, ^16^, ^17^ ^]^

**Scheme 1:**
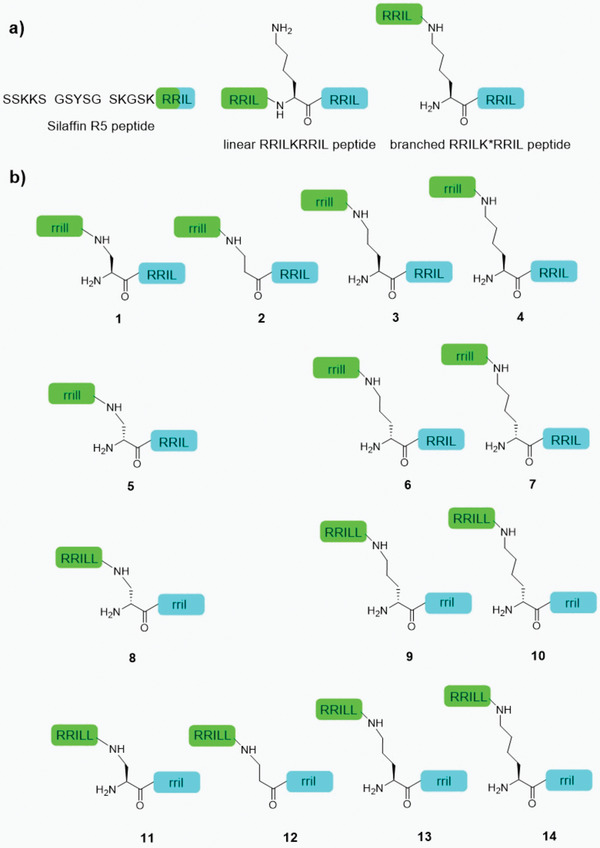
A) Overview of the R5 peptide and RRIL peptide dimers. B) Library of d‐ and l‐amino acids containing isopeptide RRIL/rrill dimers explored here. Lowercase letters indicate d‐amino acids. Central amino acids used here are l‐ or d‐Dap = 2,3‐diaminopropionic acid in peptides 1, 5, 8, and 11, βAla = β‐alanine in peptides 2 and 12, l‐ or d‐Orn = ornithine in peptides 3, 6, 9, and 13 and l‐ or d‐Lys = lysine in peptides 4, 7, 10, and 14.

Although the exact mechanism of R5‐mediated silica formation has not yet been fully revealed, it is thought to start with the self‐assembly of the R5 peptide.^[^ ^18^ ^]^ This theory is supported by the observation that the formation of uniform silica particles requires extended preincubation periods of R5 in a phosphate‐containing buffer prior to exposure to silicic acid. Sequence function analyses have shown that the C‐terminal region of R5, consisting of residues RRIL, is key for silica precipitation (**Scheme** **1**a).^[^ ^1^, ^19^ ^]^ Silica particles do not form in vitro when the RRIL motif is cut off the peptide. Interestingly, the RRIL motif is proteolytically removed in vivo, and its physiological role remains unclear.^[^ ^12^ ^]^ This circumstance is further complicated by the fact that the tetrapeptide alone is incapable of forming silica particles. The pentapeptide KRRIL, however, can induce silica precipitation, albeit with limited efficiency and leading to disordered solid phases.^[^ ^19^ ^]^

Today, various properties of the RRIL sequence have been explored, e.g., by combining two RRIL blocks to form the linear RRILKRRIL peptide (Scheme 1a). The silica‐precipitating properties of this peptide were similar to those of the R5 peptide. Other variants of RRILKRRIL, differing in stereochemistry and linkage, did not change the silica‐precipitation properties. In contrast, a dimeric variant rrill‐l‐K*‐RRIL **4** displayed dramatically shifted properties.^[^ ^20^ ^]^ Comprising only d‐amino acids and containing an extra leucine residue in the rrill motif connected via the lysyl epsilon‐amine, this peptide underwent self‐assembly resulting in large rod‐like structures in phosphate buffer at pH 7 – in contrast to the otherwise typically spherical nanoparticles. Along these lines, another variant with the sequence rrilK*RRLL, where l‐leucine in the C‐terminal motif replaces l‐isoleucine, led to the formation of sheet‐like structures. Although it is known that exposure of the peptides to silicic acid leads to a silica “coating” that constitutes the solid structures (as confirmed by electron microscopy^[^ ^21^, ^22^, ^23^ ^]^), the structure–function relationship between peptide structure and the precipitation properties remains unclear; in particular upon covalent combination of the RRILL and RRIL motifs.

The auspicious properties of RRILL‐based peptides and the outlook for rational solid‐phase design warrant a deeper investigation of these substances. Therefore, herein, we shed light on the mechanistic details of the peptide assembly and the silica‐precipitation mechanism. We devise first steps toward morphology control in RRIL‐induced peptide assembly and silica precipitation leading to design principles for solid phases. Given the ease of supramolecular variability, the dimeric RRIL system constitutes a versatile and readily accessible model system to investigate questions of peptide self‐assembly and biomimetic silica formation. Capitalizing on these features, we explore further variants of the branched rrill‐l‐K*‐RRIL **4** system by varying the central lysine residue (Scheme 1B). We show that the size and stereochemistry of this residue are essential for guiding self‐assembly. We integrate these findings with in‐depth NMR and computational analyses showing that conformational variations of soluble supramolecular preprecipitation species act as templates for peptide self‐assembly and, in turn, for silica structures in the solid. Thus, we provide synthetically robust and biocompatible routes toward new morphologies rationally designed for different applications.

## 2 Results and Discussion

To investigate the impact of the central lysine and the peptide stereochemistry on the self‐assembling properties of different KRRIL‐based constructs, 14 variants were prepared using solid‐phase peptide synthesis (SPPS, Scheme 1B). After synthesis, the peptides were purified by reverse phase high‐performance column chromatography (RP‐HPLC). The purity of the resulting products was determined via HPLC and mass spectrometry (Figures S1–S28 and Schemes S1–S14, Supporting Information), after which silica precipitation and SEM‐imaging were performed according to previously reported protocols.^[^ ^17^, ^18^, ^19^, ^20^, ^24^, ^25^, ^26^, ^27^ ^]^

**Figure 1:**
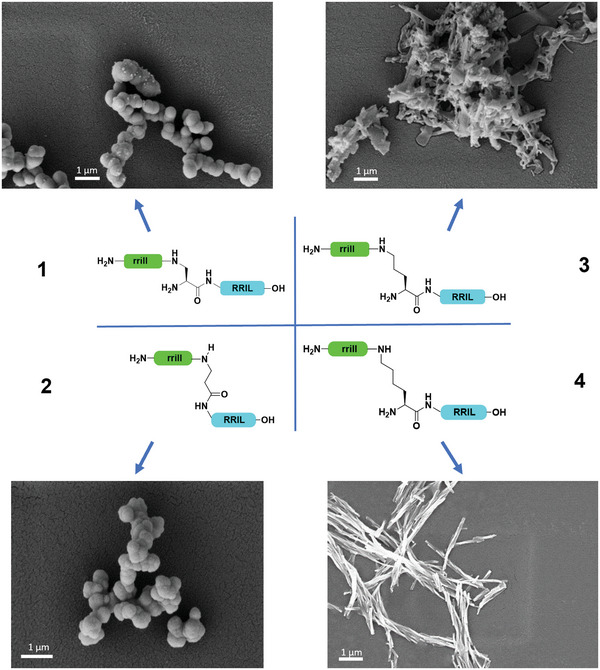
Comparison of the peptides 1–4 with respect to silica particle precipitation. The sidechain‐attached rrill (green) as well as the C‐terminal RRIL (blue) sequences and the stereochemistry of the linker are kept constant, whereas the linker length was varied by using l‐Dap, βAla, l‐Orn, or l‐Lys as central residue.

We modified two key factors previously shown to severely impact silica morphology: the sidechain length of the central residue (which acts as a linker and controls the distance between both RRIL motifs) and its stereochemistry. The impact of the distance between the rrill and RRIL motif on silica‐precipitating properties of the construct was investigated first. The variation of the distance by use of l‐diaminopropionic acid (l‐Dap—1 CH_2_ moiety), β‐alanine (βAla—1 CH_2_ moiety, no α‐amino group), or l‐ornithine (l‐Orn—3 CH_2_ moieties) instead of l‐lysine (l‐Lys—4 CH_2_ moieties) had a strong influence on the particle morphology (**Figure** **1**). The use of βAla allowed us to determine the impact of a free amino group in α‐position. However, we observed that both, l‐Dap and βAla containing peptide variants (**1** and **2**), led to spherical particles, and a direct impact of the free amino group was excluded (Figure 1; Figures S2 and S4, Supporting Information). At the same time, contrasting behavior was found between peptides rril‐l‐Dap*‐RRIL (**1**) and rrill‐l‐Orn*‐RRIL (**3**). The two additional methylene groups of the ornithine linker led to different silica precipitates with sheet‐like morphology (Figure 1; Figure S6, Supporting Information). Similarly, further extension of the linker in peptide rrill‐l‐K*RRIL (**4**) resulted in a defined rod‐like silica morphology (Figure 1; Figure S8a, Supporting Information). This finding agrees with previous experiments reported by our group.^[^ ^20^, ^22^ ^]^ Silica particles derived from l‐ornithine‐containing peptides resemble an intermediate stage in the transition from spherical morphology with l‐Dap‐ and β‐Ala as central residues to the rod‐like morphology derived from **4**. Peptides with l‐ornithine and l‐lysine as central residues showed precipitations after incubation for 30 min in phosphate buffer (in the absence of silica), a behavior not observed for peptides containing l‐Dap and β‐Ala. This behavior could be due to increased exposure of hydrophobic residues to the aqueous environment in the presence of phosphate ions, which greatly impacts the self‐assembly pathway. Even though initially considered an undesired effect, this enabled SEM analysis of the precipitates. Peptide **3** led to a sheet‐like structure (Figure S6b, Supporting Information), while peptide **4** (Figure S8b, Supporting Information) formed rod‐shaped peptide aggregates, very similar in size and shape to the silica particles shown in Figure 1 for these two peptides. This observation clearly indicates the templating effect of the peptide assemblies for silica precipitation, an event depending on phosphate ion concentration. At the same time, this finding implies specific differences in phosphate‐peptide interactions impacting self‐assembly.

**Figure 2:**
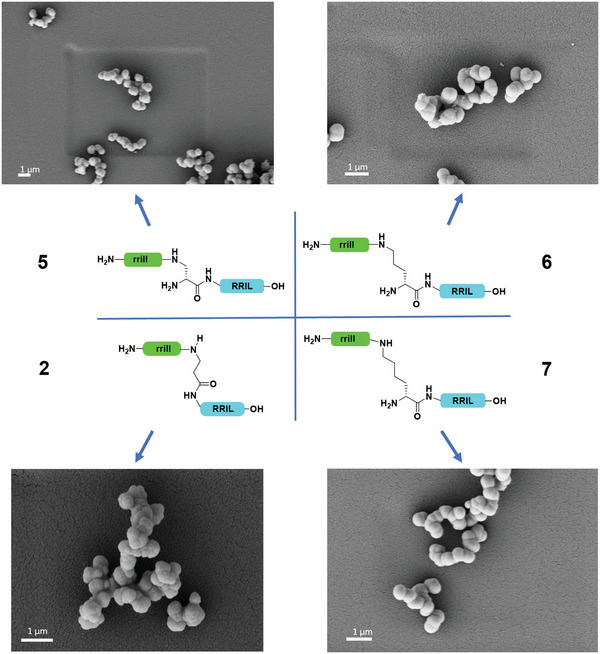
Comparison of the peptides 2, 5–7 with respect to silica particle precipitation. The sidechain‐attached rrill (green) as well as the C‐terminal RRIL (blue) sequences and the stereochemistry of the linker are kept constant, whereas the linker length was varied by using d‐Dap, βAla, d‐Orn, or d‐Lys as central residues.

A dramatically different picture emerged upon toggling the stereochemistry of the linker residues. All silica particles obtained with peptides rrill‐d‐Dap‐RRIL **5**, rrill‐d‐Orn‐RRIL **6**, and rrill‐d‐K*‐RRIL **7** showed a spherical morphology (with similar size distribution) as typically found for R5 and peptides **1** and **2** (**Figure** **2**). Noteworthy, when switching from l‐ to d‐Lysine (**4** and **7**), the silica particle morphology shifted entirely from rod‐like to spherical. Recent studies have shown that replacing l‐lysine with d‐lysine can significantly change the conformational propensity of adjacent amino acids in short peptides.^[^ ^28^ ^]^ This effect may cause the strongly deviating morphology here, too, particularly when considering the switch in orientation of the rrill and RRIL motifs towards each other when going from l‐ to d‐amino acids in the central position. Aggregation propensities also vary between peptides with l‐ or d‐ornithine and lysine. The d‐variants (**6** and **7**) did not show any precipitation after incubation with phosphate buffer, indicating a different self‐assembly process.

**Figure 3:**
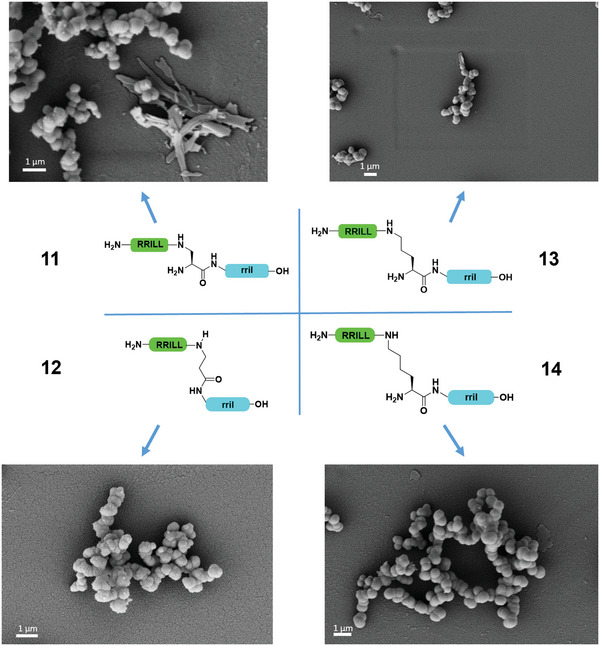
Comparison of peptides 11–14 with respect to silica particle precipitation. The sidechain‐attached RRILL (green) as well as the C‐terminal rril (blue) sequences and the stereochemistry have been switched to generate the enantiomers of peptides 5, 6, and 7.

Mirror images of peptides were investigated next, with the rrill and RRIL motifs switched from C‐terminus to sidechain, and vice versa. As expected, the particles resulting from enantiomers of **5**, **6**, and **7** (**11**, **13**, **14,** respectively) displayed the same spherical morphology (**Figure** **3** and Supporting Information), indicating a similar self‐assembly mechanism. Again, instead of rod‐like particles, spherical silica particles were formed during precipitation with peptide **14**, with d‐lysine as a central residue. Therefore, the stereochemistry of the lysine linker from the adjacent leucine appeared as another key factor for obtaining silica particles with a rod‐like morphology. Interestingly, peptide **11** formed a mixture of spheres and rods.

**Figure 4:**
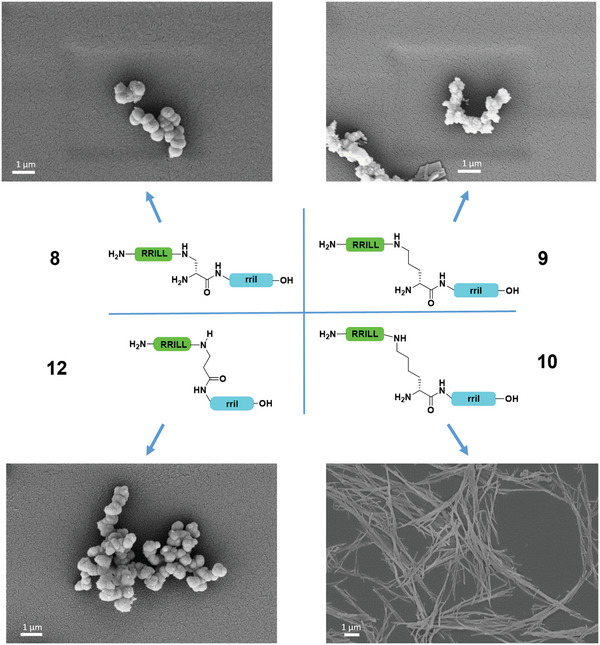
Comparison of peptides 8–10 and 12 with respect to silica particle precipitation. The sidechain‐attached RRILL (green) as well as the C‐terminal rril (blue) sequences and the stereochemistry have been switched to generate the enantiomers of peptides 1, 3, and 4.

Similarly, enantiomers of peptides **1**, **3**, and **4** (**8, 9**, and **10**, respectively) showed the same behavior (**Figure** **4**; Supporting Information), also during peptide precipitation in phosphate buffer in the presence of peptides **9** and **10** (Figures S18b and S20b, Supporting Information). The particles containing ornithine, for example, showed a strong similarity to the particles of the enantiomer equivalent, and the rod‐like morphology was again observed for the lysine‐containing peptide **10**. We assume that the dramatic change in particle morphologies is directly induced by the stereochemistry of the central lysine residue. Indeed, the difference between peptides **4** and **7** as well as **10** and **14** lies only in the exchange of l‐ and d‐lysine.

To understand the relations between the chemical structures and varying self‐assembly behaviors of our rrillK*RRIL variants, we employed solution‐state nuclear magnetic resonance (NMR) spectroscopy in combination with molecular dynamics (MD) simulations.

**Figure 5:**
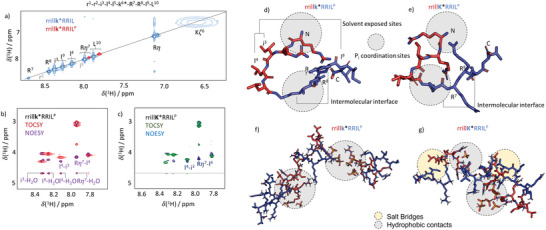
a) H^N^‐region of ^1^H‐^1^H TOCSY spectra of rrillk*RRIL **7** in water (blue) and PBS (red). The resonance assignment is indicated. The resonance of K^6^ is heavily broadened due to rapid proton exchange. Dissolution in PBS leads to a disappearance of most resonances. b) H^N^/H^α^‐region of ^1^H‐^1^H TOCSY (red) and NOESY (purple) spectra of **7** in PBS. Residues i^3^, l^4^, I^9^, and the side chain of R^7^ indicate solvent exposure through NOE and/or exchange‐based cross‐peaks with the water resonance. c) H^N^/H^α^‐region of ^1^H—^1^H TOCSY (red) and NOESY (purple) spectra of rrillK*RRIL **4** in PBS. In contrast to **7**, no water interaction can be observed. d) Simulated structure of 7 in PBS. The l‐ and d‐blocks are indicated by the red/blue color code. N‐ and C‐termini are labeled. Note the hinge involving the k^6^ isopeptide bond. e) Simulated structure of **4** in PBS. f) Aggregate structure found in MD simulations of 5 copies of **7** in PBS. The gray circles indicate salt bridges formed by the phosphate ions and positively charged arginines. g) Same as in panel (f), but for **4**. Besides the salt bridges, hydrophobic side‐chain contacts (yellow circles) cause compaction of the aggregates.

Due to differences in precipitate morphologies being most pronounced, we focused on the contrast between the rod‐like precipitates formed by rrill‐l‐K*‐RRIL **4** and the spherical precipitates by rrill‐d‐k*‐RRIL **7**. At first, we assigned all backbone amide ^1^H^N^ resonances utilizing total correlation spectroscopy (TOCSY) and correlation spectroscopy (COSY) (**Figure** **5**a; Figures S29 and S30, Supporting Information). Next, we dissolved the peptides in a phosphate‐buffered aqueous solution at pH 7; as used in the silica‐precipitation assays. We observed that most of the resonances disappeared upon exposure to phosphate ions (Figure 5a). Only signals of the C‐terminal residues (I^9^ and L^10^) remained strongly above the detection threshold. All other signals were either invisible or very weak. For both peptides **4** and **7**, this observation points towards the assembly (aggregation) of the peptides upon dissolution in phosphate buffer, causing a reduction in residue mobility and broadening of the NMR signals beyond the detection threshold. As the C‐terminal residues, I^9^ and L^10^ remained detectable, a morphology in which the C‐termini constitute the solvent‐accessible surface (SAS) of the self‐assemblies appears very likely since surface amino acids are less restricted in terms of residue mobility.

Interestingly, nuclear Overhauser spectroscopy (NOESY) showed pronounced differences between the two peptide assemblies. **7** displayed various strong cross‐peaks between the water resonance and the peptide amides (Figure 5b). In contrast, **4** did not lead to the observation of any strong correlations with the solvent (Figure 5c). Hence, the NMR data suggest that the switch between the d‐ and l‐configured central lysine residue changes the supramolecular configuration. The intense water‐peptide cross‐peaks indicate a lesser aggregation density of **7** compared to **4**. This finding hints at an explanation of the observed differential self‐assembly behaviors. It should be noted that the absence of water‐amide cross‐peaks can furthermore indicate the formation of secondary structure elements, in which all ^1^H^N^ nuclei are involved in hydrogen‐bond networks that impede solvent interactions. However, the H^α^ chemical shifts did not change upon self‐assembly (Figure S46, Supporting Information), which would point towards high secondary structure propensities.

To further deepen our understanding, we integrated the NMR data with molecular dynamics (MD) simulations. To this end, we predicted the structures of the rrill and KRRIL blocks using PEPstrMOD^[^ ^29^, ^30^ ^]^ and formed the iso‐peptide bond between d‐Ile and d‐Lys and d‐Lys, respectively, in silico to create a starting structure for our simulations. The structures were simulated in explicit solvent for 700 ns for both peptides in the presence of phosphate ions. The structures after 700 ns are shown in Figure 5d,e. We could infer that the l‐configured variant led to conformational compaction not observed for the d‐variant (an analysis of dynamic cross‐correlation matrices further shows this; see Figure S31 in the Supporting Information). Hence, the stereochemical switch possibly causes compaction of **4,** which would explain the missing TOCSY water cross‐peaks for this peptide. Subsequently, five copies of both structures, respectively, were randomly placed in boxes loaded with 50 × 10^−3^ m sodium phosphate in explicit water. The resulting starting configurations were then propagated for another 700 ns (Figures S32–S35, Supporting Information). Within 200 ns, the peptides started aggregating. Figure 5f,g shows the structures obtained again after 700 ns. Two important observations were made in this context: (i) the negatively charged phosphate ions mediate the intermolecular attraction of the peptides by forming ionic bridges between the positively charged arginine residues (gray circles in Figure 5f,g; ^31^P‐detected NMR data supporting the direct interaction between the peptides and the P_i_ counterions can be found in Figures S40 and S41 in the Supporting Information). Note that the pKa value of the arginine guanidinium group is 13.8. Hence, we assume all Arg‐sidechains to be positively charged.^[^ ^31^ ^]^ (ii) Peptide **7** formed elongated assemblies compared to **4,** indicating that the l‐configuration causes compaction not only of the monomer building blocks but also of the supramolecular self‐assemblies.

These observations are supported by earlier reports of MD simulations by Lutz et al.^[^ ^32^ ^]^ and Sprenger et al.,^[^ ^33^ ^]^ who showed that the RRIL motif is key to the intermolecular interactions of the R5 peptide underlying the design of **4** and **7** and that the presence of phosphate moieties is essential for the functioning of the RRIL motif. Together with other reports,^[^ ^34^, ^35^, ^36^ ^]^ these data strongly suggest the feasibility of MD simulations to study the interaction and aggregation of R5 in silico. However, it should be noted that it cannot be excluded that the structures shown in Figure 5 are trapped in local energetic minima due to the finite length of the MD trajectories. The total energy minimum might therefore differ from the shown conformations. Hence, a stand‐alone analysis of the MD data alone should be considered with care, although replicas of the MD trajectories (Figures S42 and S43, Supporting Information) reproduced the results twice. Nonetheless, the computational data aligns well with the experimental NMR data and rationalizes the NMR spectra. The integrative analysis of both experimental and computed data led to the model presented in the following paragraph.

Our simulations suggest additional attraction between the hydrophobic side chains of the isoleucine and leucine residues as the underlying cause for the switch in silica particle morphology (Figure 5g; cf. DCCM in Figure S36 in the Supporting Information). Four NMR observations support this finding. (i) the more expanded **7** provides a larger SAS (i.e., more water cross‐peaks in the NOESY data) than its counterpart **4**. (ii) The NOESY spectrum of **4** shows significantly more cross‐peaks between the hydrophobic side chains and the backbone of the peptide (Figure S37, Supporting Information). (iii) The observation of a stronger aggregation tendency for **4** was further confirmed by diffusion‐ordered spectroscopy (DOSY) that showed an average hydrodynamic radius of 0.30 nm for assemblies of **7** and 1.01 nm for **4** (Figure S38, Supporting Information). In the absence of any phosphate, i.e., in neat water, both peptides consistently showed an *R*_h_ of 0.23 nm, corresponding to the respective monomers with identical molar mass.

(iv) In line with the TOCSY spectra, the MD simulations show that the C‐termini of both peptides tend to point towards the solvent confirming that the SAS of the aggregates is formed by the C‐terminal residues of the peptide derivatives. Additional evidence for the importance of the hydrophobic contacts of Ile 9 in peptide **4** is provided by a more hydrophilic peptide variant, in which Ile 9 was replaced by Gln (peptide **4′**, for analytical data see Figure S7C,D in the Supporting Information). This peptide also precipitates silica but leads to spherical particles (Figure S8c, Supporting Information), providing additional experimental proof that hydrophobic contacts in position 9 are required for rod formation (Figure S8c, Supporting Information) .

Considering that **4** formed rods and **7** spherical particles upon exposure to silica, it can now be stated that the precipitation event takes off from different templates formed in solution. Hence, the shapes of the solid morphologies are already determined beforehand through the configurations of the precursors formed in the presence of phosphate ions. The additional hydrophobic side‐chain contacts found for **4** act as a structure‐guiding force that compacts the self‐assemblies into defined arrays and forces the aggregates into rod‐like assemblies. In contrast, spherical particles are formed when electrostatic intermolecular forces act alone, as in **7**. This observation is potentially a result of long‐range electrostatic interactions that are often non‐directional,^[^ ^37^ ^]^ particularly when not embedded in more complex peptide folds.

**Figure 6:**
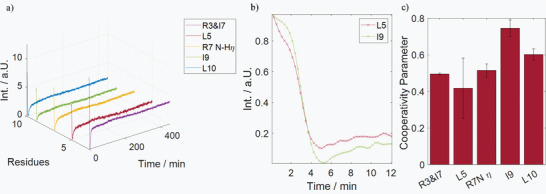
Real‐time monitoring of 4 precipitation. a) Residue‐resolved intensity time‐traces after addition of the silicic acid. Initially, the signals lose intensity due to the precipitation event. After ≈15 min all resonances reappear, pointing towards a weak resolubilization effect. b) Comparison of the initial intensity decay for residues L^5^ and I^9^. The C‐terminal isoleucine loses its signal slower than the central leucine. c) Cooperativity parameter for all visible residues as determined by fitting a sigmoidal function to the first 5 min of the time traces in (a). The higher the value, the slower the onset of the precipitation event. Residues I^9^ and L^10^ show higher values, i.e., a slower signal loss.

To confirm that the structure of the precursors in PBS are indeed acting as templates for the solid phase morphologies and that their conformational features are translated from the solution to the solid‐state, we also investigated the silica coprecipitation of **4** by real‐time NMR (**Figure** **6**). To this end, we recorded a ^1^H spectrum every 15 s after the addition of silicic acid to the precursor solution. Due to the sparseness of the spectra in phosphate buffer, residue resolution could be achieved even by 1D detection. We traced the signal amplitudes of all residues that remained above the detection threshold over time. Figure 6a,b shows the resulting intensity curves. The formation of the peptide‐silica precipitates led to a complete signal loss within 5 min for all residues. An analysis of the precipitation rate is shown in Figure 6c via the cooperativity parameter σ of sigmoidal fits to the data in Figure 6a (see Figure S39 for the fits in the Supporting Information). The larger σ, the slower the precipitation. We observed that residues I^9^ and L^10^ lose their signal intensities much slower than all other detectable residues (Figure 6b shows the difference between L^5^ and I^9^). This observation confirms that the SAS formed in the precursors by residues I^9^ and L^10^ remains intact even throughout the initial phase of the precipitation event. Along these lines, it can be observed that the overall intensity pattern, i.e., strong C‐terminal signals versus weak signals from all other residues, is maintained throughout the precipitation process. Hence, the features observed in the solution‐state precursors are retained throughout the silica precipitation.

Two further points can be noted

i) The differential precipitation kinetics, i.e., the slower signal decay of residues I^9^ and L^10^ compared to the other residues, point toward a complex precipitation mechanism that involves at least two stages. First, the templates take part in the formation of larger particles that yet stay in solution since I^9^ and L^10^ remain detectible by solution‐state NMR even though all other signals are already below the sensitivity threshold. Secondly, these soluble peptide‐silica “meso”‐particles associate to form the solid phase. We already reported similar behavior for liquid‐to‐solid transitions of stimuli‐responsive polymers.^[^ ^38^, ^39^ ^]^
ii) All signals reappear weakly 15 min after initiation of the precipitation event, pointing towards weak resolubilization of the peptides. This observation agrees with the release of peptides from silica precipitates previously described by us and others.^[^ ^40^, ^41^ ^]^

## 3 Conclusions

By exploiting the capacity of the KRRIL motif to guide silica into defined solid morphologies under mild conditions in vitro, we developed a portfolio of rrill‐X*RRIL‐ and RRILL‐X*rril‐type peptides that enables control over the shape of the silica particles. We show that by systematically varying the length and stereochemistry of the linker residue X, rational design of solid phases becomes possible.

We rationalized this control over the morphology by manipulating the linker residue and showing that the structure of supramolecular precursors, i.e., templates determines the shape of the final silica particles. Indeed, the structural features of the templates translate from the solution state to the solid phase. Notably, we find that template density and size, as determined by the interplay between electrostatic and hydrophobic forces, critically impact the silica morphology. As a first step towards a design principle, it may thus be stated that the precursor architecture needs to be toggled to control the solid phase. In particular, hydrophobic interactions, enabled by using a d‐configured lysine, guide the precipitates into rod‐like morphologies. Other linker types that favor only nondirectional electrostatic interactions as a structure‐guiding force led to the formation of spherical particles. Interestingly, our data also show that the peptides form the inner scaffold of the particles, which is only coated with silica upon exposure. This points towards a very defined supramolecular architecture of the peptide self‐assemblies that persists within the particles. Note that the formation of soluble precursors that determine the fate of solid phases upon precipitation has been observed for purely inorganic biomineralization events, too. In the context of nonclassical nucleation theory^[^ ^42^, ^43^, ^44^ ^]^ it was recently shown that the preformation of templates can precisely guide precipitation pathways and can be exploited for rational materials design. The same concept is transferred here to peptide‐based biomineralization mimicry. The here‐presented RRIL‐based opportunities for morphology control translate these concepts to guide solid‐phase formation through designing precursors to hybrid peptide‐silica particles and thereby open an avenue for creating novel materials with tailored properties.

## 4 Experimental Section

All peptide syntheses described below were performed according to Fmoc strategy using Wang‐resins as a solid phase.^[^ ^45^ ^]^ The synthesis steps were carried out on a 0.01 mmol scale and at room temperature unless otherwise stated. The washing steps were carried out with ≈1 mL DMF per 25 mg resin.

First, the Wang‐resin was weighed in a syringe with frit and swollen with DMF for 45 min. The Fmoc deprotection was then performed with 20% piperidine in DMF solution with first 3 min and second 7 min reaction time (1 mL/25 mg resin). Afterwards the resin was washed five times with 1 mL DMF. For the coupling of native amino acids 5 eq of Fmoc‐protected amino acid were dissolved in 4.2 eq HATU (0.5 m HATU solution in DMF), 10 eq DIPEA were added and the mixture was activated for 3 min at RT. The coupling solution was then transferred to the resin with a reaction time of 30 min. After that, the resin was filtered and washed 10× with 1 mL DMF. The cycle started again with Fmoc deprotection. For overnight storage, the resin was kept in DMF.

Before purification, a HPLC‐MS run of the crude product was performed on an analytical Kromasil C4 RP‐HPLC column using a gradient from 5% to 45% buffer B (ACN with 0.08% TFA) in buffer A (H_2_O with 0.1% TFA) over 20 min at a flow rate of 1 mL min^−1^. The crude peptide was then dissolved in 8 mL deionized water and injected for purification on Waters Prep 150 System on a Kromasyl C4 semipreparative RP‐HPLC column using a gradient from 5% to 45% buffer B in buffer A over 30 min at a flow rate of 10 mL min^−1^. The fractions of all UV peaks with an absorption above 100 mAU were collected automatically. Subsequently, 20 µL of each fraction were directly injected into Thermo Fisher HPLC‐MS system to identify product peaks based on mass spectra. The fractions in which a product peak without impurities was identified were combined and lyophilized overnight.

For the final analysis via RP‐HPLC a small amount of the purified peptide was dissolved in buffer A, injected into a Dionex Ultimate 3000 HPLC system and separated by an analytical Kromasil C4 RP‐HPLC column using a gradient from 5% to 65% buffer B in buffer A over 30 min at a flow rate of 1 mL min^−1^. In a further step, a small amount of peptide was dissolved in water and directly injected into a Thermo Fisher HPLC‐MS system.

### Silica Precipitation

First, a 4 mg mL^−1^ solution of purified peptide (≈3 × 10^−3^ m) in deionized water was prepared. Then the peptide solution was diluted 1:1 with 100 × 10^−3^ m potassium phosphate buffer (pH 7), so that the final peptide concentration was 2 mg mL^−1^. The solution was then incubated for 24 h at RT. Sometimes a precipitate occurred after incubation with phosphate buffer, which, unless otherwise stated, was not separated. Next day, silicic acid was freshly prepared by adding 40 µL (270 µmol) tetramethoxysilane to 960 µL 1 × 10^−3^ m HCl, vortexing for 3 s and incubating for 4 min at RT. Then 10 µL from the resulting silicic acid solution were added to 100 µL of the 2 mg mL^−1^ peptide solution in potassium phosphate buffer, so that the final concentration of silicic acid was 25 × 10^−3^ m. The solution was then vortexed and incubated for 30 min at RT to initiate silica precipitation. After precipitation the suspension was centrifuged (RT, 5 min, 14 000 rpm) and the precipitate was washed twice with 100 µL H_2_O each. Finally, the precipitate was resuspended in 1 mL of H_2_O for the preparation of SEM samples.

### Scanning Electron Microscopy of Peptide Precipitates

The precipitate that formed upon incubation of peptide in phosphate buffer was spun down, separated and resuspended with 1 mL 50 mmol phosphate buffer (pH 7). 3 µL of the resulting suspension were applied to a ThermanoxTM coverslip and air‐dried. The sample applied to the coverslip was then washed three times with 3 µL H_2_O and dried. A layer of gold was subsequently applied to all samples by sputter coating under high vacuum (Bal‐Tec SCD 005) and SEM imaging was performed with a Zeiss SEM supra 55 VP at 20 kV.

### Scanning Electron Microscopy Imaging of Silica Particles

10 µL of the silica particles suspension from Section 2.9. were applied onto ThermanoxTM coverslips and dried on air. A layer of gold was subsequently applied to all samples by sputter coating in high vacuum (Bal‐Tec SCD 005) and SEM imaging was performed with a Zeiss SEM supra 55 VP at 20 kV.

### Nuclear Magnetic Resonance Spectroscopy

For NMR spectroscopy, peptides were dissolved at 1 mg mL^−1^ in neat water for signal assignment or 50 × 10^−3^ m PBS buffer at pH 7.0 (90% H_2_O and 10% D_2_O; 25 × 10^−3^ m Na_2_HPO_4_, 25 × 10^−3^ m KH_2_PO_4_, 25 × 10^−3^ m NaCl) for the precipitation assays. NMR spectra were recorded on a 600 MHz Bruker NEO spectrometer equipped with a Prodigy TCI probe head. All spectra were acquired at 25 °C.

TOCSY and NOESY data were recorded using the Bruker’ dipsi2gpph19’ and “noesyfpgpphrs19” pulse sequences for TopSpin 4. All TOCSY spectra were recorded with a spectral width of 8196.7 Hz in both dimensions and 32 scans. The mixing time was 150 ms. QUADRATURE detection was done using States‐TPPI. All NOESY spectra were measured with a spectral width of 9615.3 Hz in the F2 dimension and 7202.1 Hz in the F1 dimension. 32 scans were recorded. The mixing time was 300 ms. QUADRATURE detection was employed again using the States‐TPPI sampling scheme. Spectral processing was achieved using TopSpin 4, NMRPipe,^[^ ^46^ ^]^ and Sparky.^[^ ^47^ ^]^ All data were zero filled to twice the original number of data points and apodized using a 60° shifted sine bell function prior to Fourier transformation. This was followed by a polynomial baseline correction in the frequency space.

For DOSY the “stebpgp1s19” pulse program of the Bruker TopSpin 4.0 pulse program library was used. 64 data points with different z‐gradient strengths were recorded. The *z*‐gradient was varied linearly between 0 and 0.1 T^2^ m^−2^. The diffusion delay was set to 800 ms. Data were analyzed using GNAT.^[^ ^48^ ^]^ Then methyl signals at 0.7 ppm were chosen for integration, and the resulting integral area versus gradient strength curve was fitted to the Stejskal–Tanner equation.^[^ ^49^ ^]^ Hydrodynamic radii were then extracted using the Stokes–Einstein law under the assumption of a spherical diffusion model.^[^ ^50^, ^51^, ^52^ ^]^

### Molecular Dynamics Simulations

Generally, the strategy outlined in Zoltan and Che et al.^[^ ^53^, ^54^, ^55^ ^]^ for simulating non‐canonical peptides was followed to generate a starting structure: Structure prediction of the d‐ and l‐blocks using PEPstrMOD, followed by the formation of the iso‐peptide bond using the YASARA software package.^[^ ^56^ ^]^ The resulting structures were then subject to energy minimization and simulated annealing in explicit solvent (atom speed <  2200 m s^−1^; 1% NaCl in water at pH 7.4 and 50 × 10^−3^ m sodium phosphate). Finally, trajectories were recorded at 25 °C at 2 fs time steps for >500 ns. From this simulation, the minimum energy structure was chosen, and 5 copies were randomly placed in a box filled with 1% NaCl and 50 × 10^−3^ m sodium phosphate in explicit water, again at 2 fs time steps for >500 ns.

Then again, MD simulations were performed using the YASARA software package.^[^ ^56^, ^57^, ^58^, ^59^, ^60^, ^61^ ^]^ The AMBER14N force field was employed with periodic boundary conditions.^[^ ^62^ ^]^ Nonbonded interactions were cut off at 1.05 nm. Long‐range Coulomb interactions were treated by a smoothed particle‐mesh Ewald method.^[^ ^63^ ^]^ Noncanonic amino acids were built and semi‐quantum‐mechanically parameterized (YAPAC‐AM1). MD trajectories of >500 ns length were accumulated for all systems. Intermolecular forces were recalculated at every second simulation sub‐step. Each production run was preceded by a short steepest descent minimization (again atom speed < 2200 m s^−1^) followed by 500 steps of simulated annealing (equilibration phase). A Berendsen thermostat was employed and barostat (NPT ensemble) coupled to the time‐averaged temperature and density, respectively, as implemented in YASARA. Temperature rescaling was employed with a set temperature of 25 °C. The average box dimensions over the last 10% of each trajectory were cubic of 36.22 Å side length (cubic cell) for one unit, cubic of 61.45 Å side length for five units (cubic cell). The cell dimensions were adjusted to yield a solvent pressure of 1 bar. Snapshots of the simulations were taken every 10 000 fs.

## Conflict of Interest

The authors declare no conflict of interest.

## Supporting information

Supporting Information

## Data Availability

The data that support the findings of this study are available from the corresponding author upon reasonable request.
