# Supplementary material for: Understanding Self‐Assembly of Silica‐Precipitating Peptides to Control Silica Particle Morphology
Source: Adv Mater. 2023 Jan 25;35(11):2207586. doi: 10.1002/adma.202207586 (PMC11475327; doi:10.1002/adma.202207586)
Supplement: Supplementary file 1 — Supporting Information [file ADMA-35-2207586-s001.pdf]

ADVANCED MATERIALS

Supporting Information

for *Adv. Mater.,* DOI: 10.1002/adma.202207586

Understanding Self-Assembly of Silica-Precipitating Peptides to Control Silica Particle Morphology

Johannes Strobl, Fanny Kozak, Meder Kamalov, Daniela Reichinger, Dennis Kurzbach, and Christian FW Becker**

Supporting Information

Understanding Self-Assembly of Silica-Precipitating Peptides to Control Silica Particle Morphology

Johannes Strobl, Fanny Kozak, Meder Kamalov, Daniela Reichinger,

Dennis Kurzbach, Christian FW Becker

Materials & Methods

Fmoc-Leu-Wang resin, Fmoc-Ile-OH, Fmoc-D-Ile-OH, Fmoc-Leu-OH, Fmoc-D-Leu-OH, Fmoc-Arg(Pbf)-OH, Fmoc-Lys(Boc)-OH, Fmoc-β-Ala-OH and (1-[Bis(dimethylamino)methylene]-1H-1,2,3-triazolo-[4,5-b]-pyridinium 3-oxide hexafluorophosphate (HATU) were purchased from Novabiochem (Nottingham, United Kingdom).

Fmoc-D-Leu-Wang resin, Fmoc-D-Arg(Pbf)-OH, Boc-L-Dap(Fmoc)-OH, Boc-D-Dap(Fmoc)-OH, Boc-L-Orn(Fmoc)-OH and Boc-D-Orn(Fmoc)-OH were obtained from Iris Biotech GmbH (Marktredwitz, Germany), Boc-Lys(Fmoc)-OH from Orpegen Peptide Chemicals GmbH (Heidelberg, Germany), Boc-D-Lys(Fmoc)-OH from Bachem Holding (Bubendorf, Schweiz). Acetonitrile (ACN) was purchased from Biosolve (Valkenswaard, the Netherlands), TFA and DCM from VWR International (Radnor, Pennsylvania USA). All other chemicals used were obtained from Sigma-Aldrich (Taufkirchen, Germany).

Analytical data of synthetic peptides

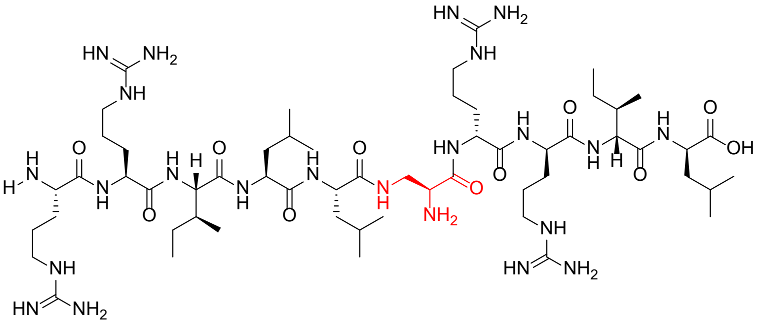

Scheme S1: Structure of peptide **1** with L-Dap as central residue. We obtained 11.6 mg (90 % for a 0.01 mmol synthesis scale).

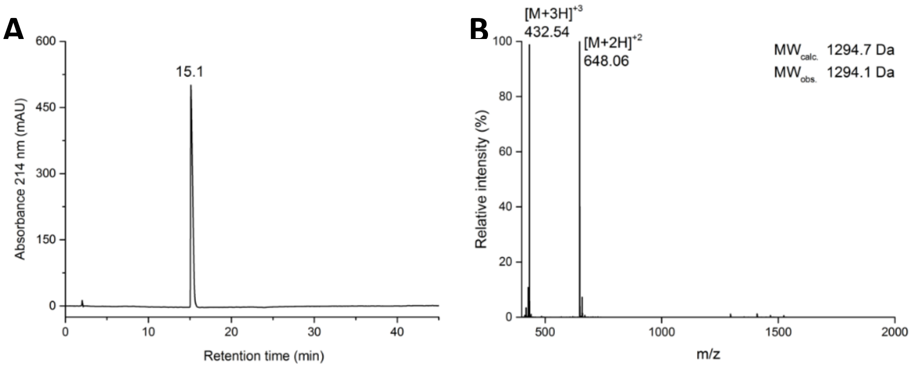

Figure S1: A: HPLC chromatogram of purified peptide **1**. B: Mass spectrum obtained from a direct injection of purified peptide 1 on a Thermo Fisher HPLC-MS system.

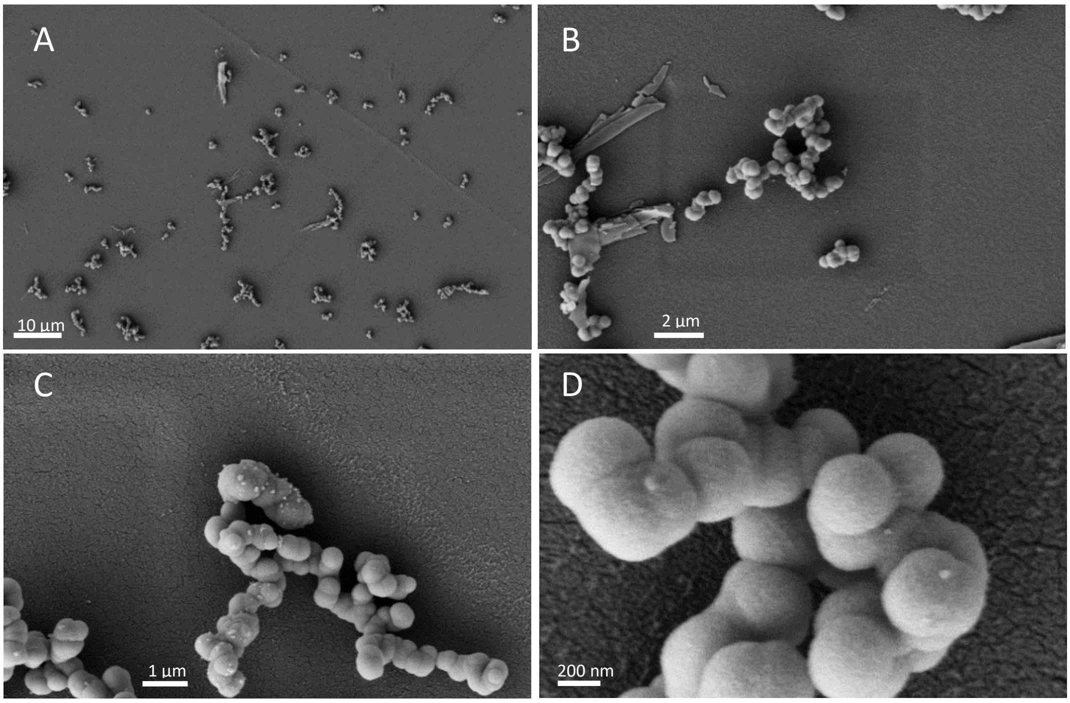

Figure S2: A-D) Selection of different SEM images of particles obtained after silica precipitation with peptide **1**.

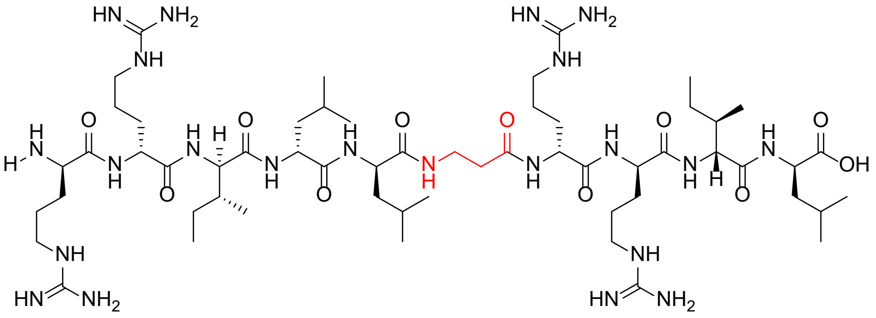

Scheme S2: Structure of peptide **2** with β-Alanine as central residue. We obtained 3.8 mg (30 % for a 0.01 mmol synthesis scale).

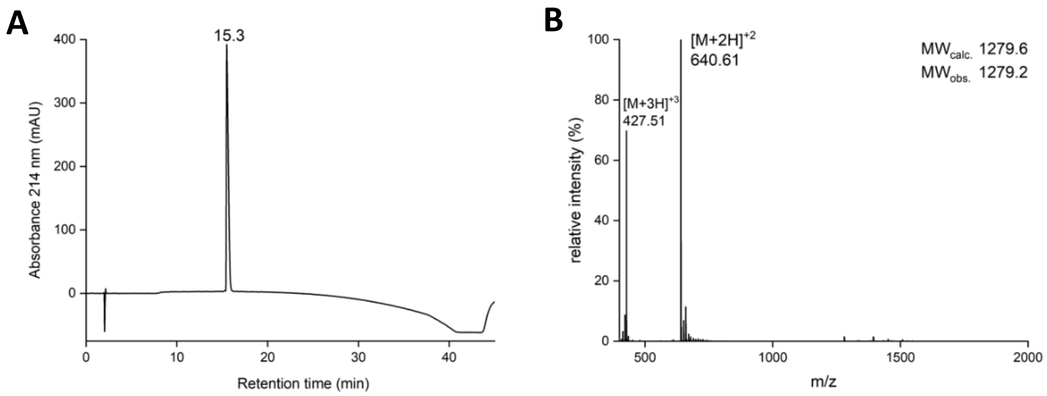

Figure S3: A: HPLC chromatogram of purified peptide **2**. B: Mass spectrum of purified peptide **2**.

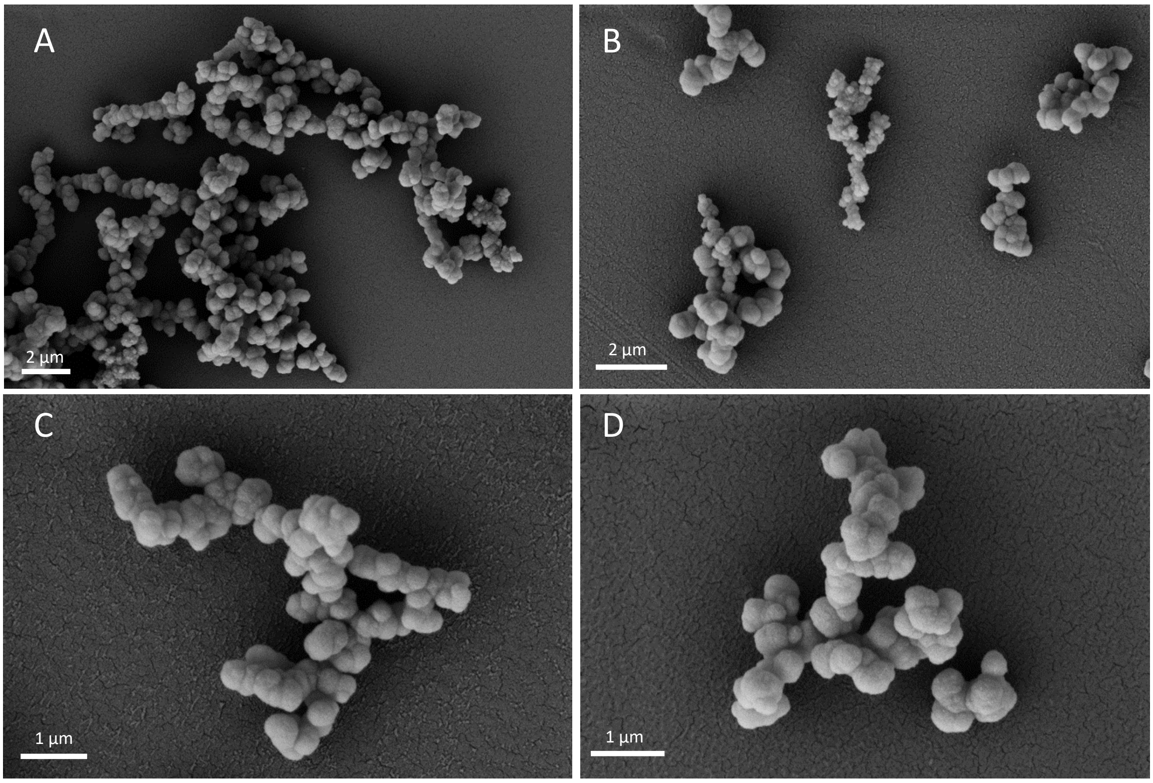

Figure S4: A-D) Selection of different SEM images of particles obtained after silica precipitation with peptide **2**.

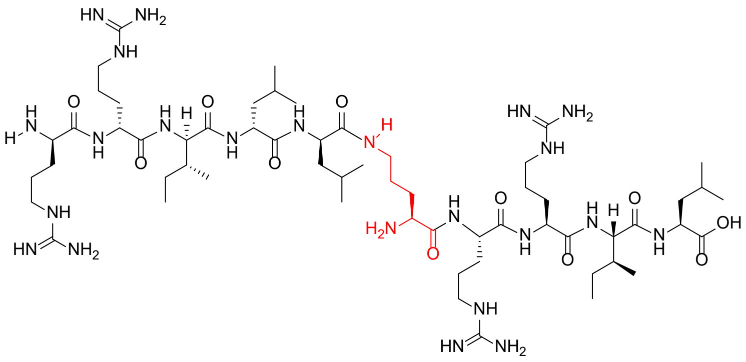

Scheme S3: Structure of peptide **3** with L-Ornithine as a central residue. We obtained 10.9 mg (83 % for a 0.01 mmol synthesis scale).

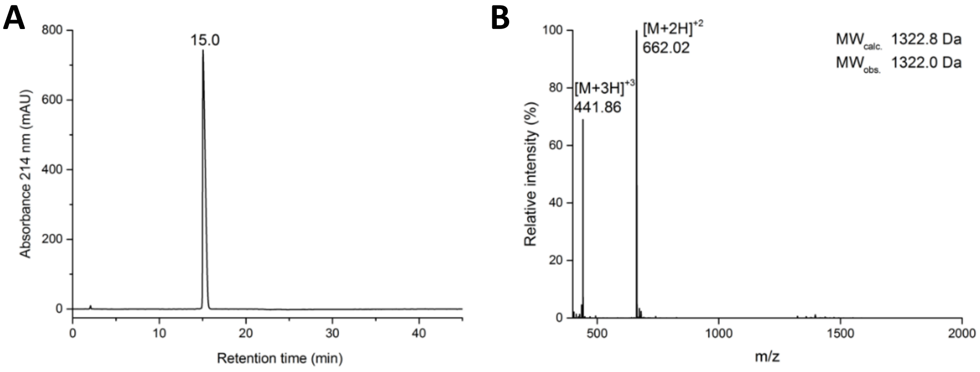

Figure S5: A: HPLC chromatogram of purified peptide **3**. B: Mass spectrum of purified peptide **3**.

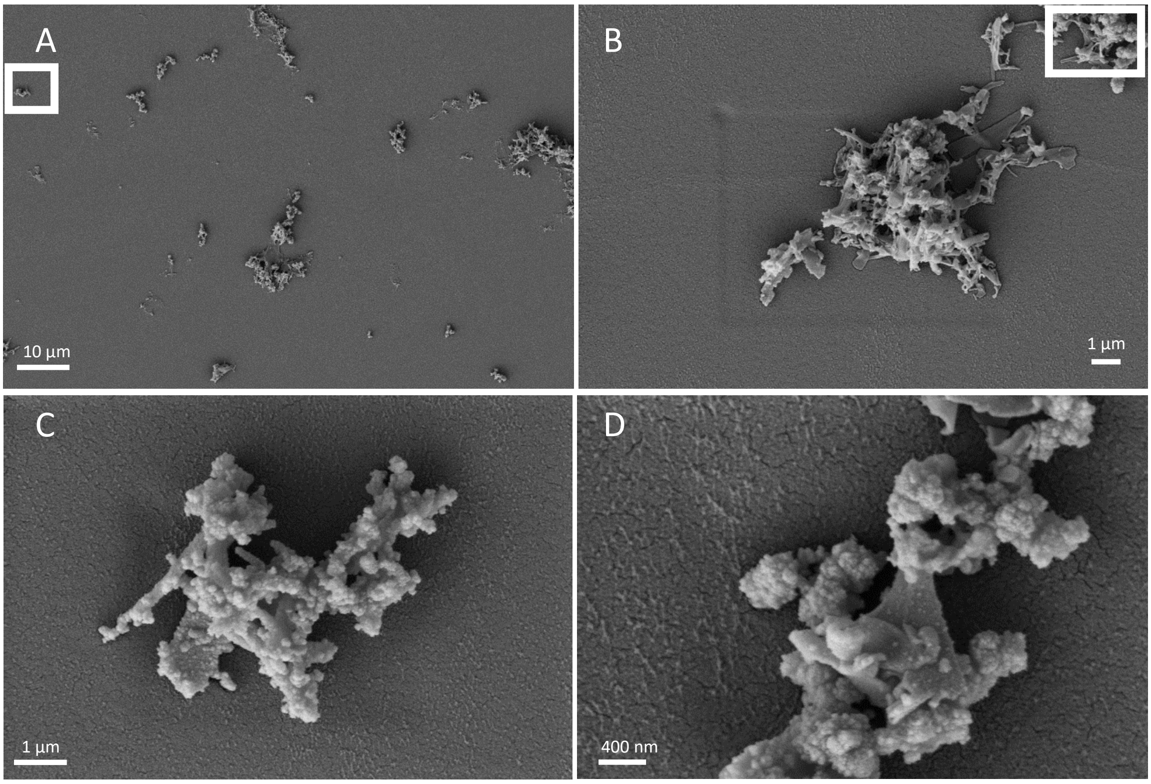

Figure S6a: A) SEM images of particles obtained after silica precipitation with peptide **3**. B) SEM images of particles obtained after silica precipitation with peptide **3** at higher magnification. C) Zoom-in to the marked area in panel A). D) Zoom-in to the marked area in panel B).

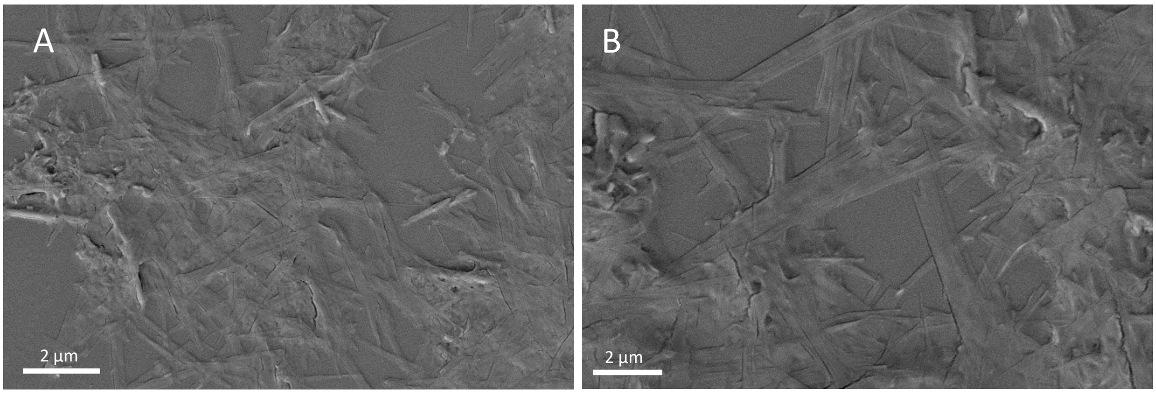

Figure S6b: A-B) Selection of different SEM images of peptide **3** precipitate obtained during incubation with phosphate buffer.

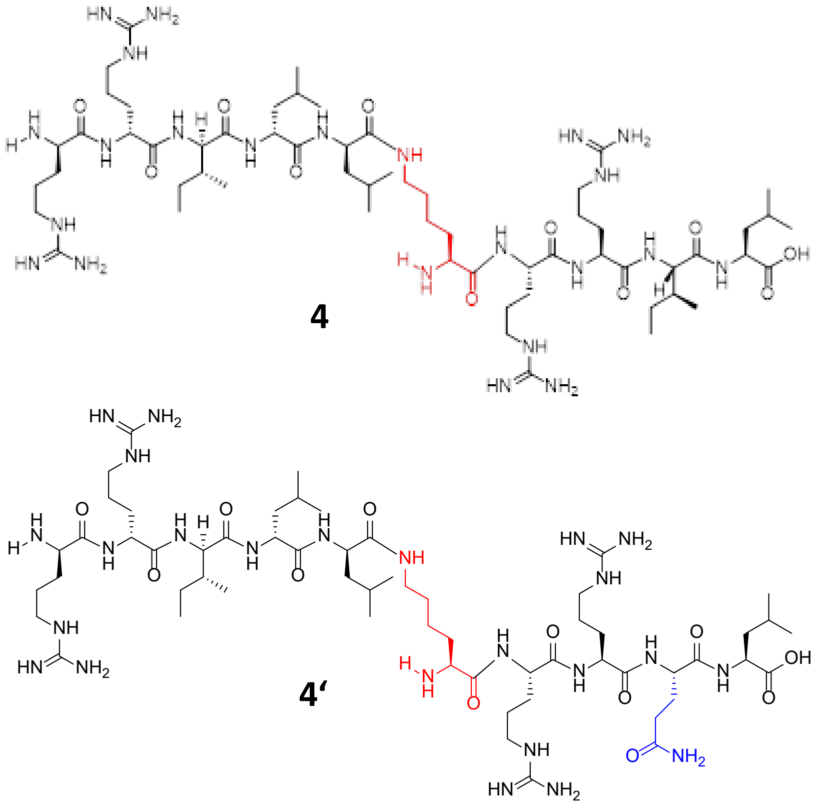

Scheme S4: Structure of peptide **4** with L-Lysine as a central residue. We obtained 12.2 mg (91 % for a 0.01 mmol synthesis scale). Peptide **4’** with Ile9 replaced by Gln (shown in blue).

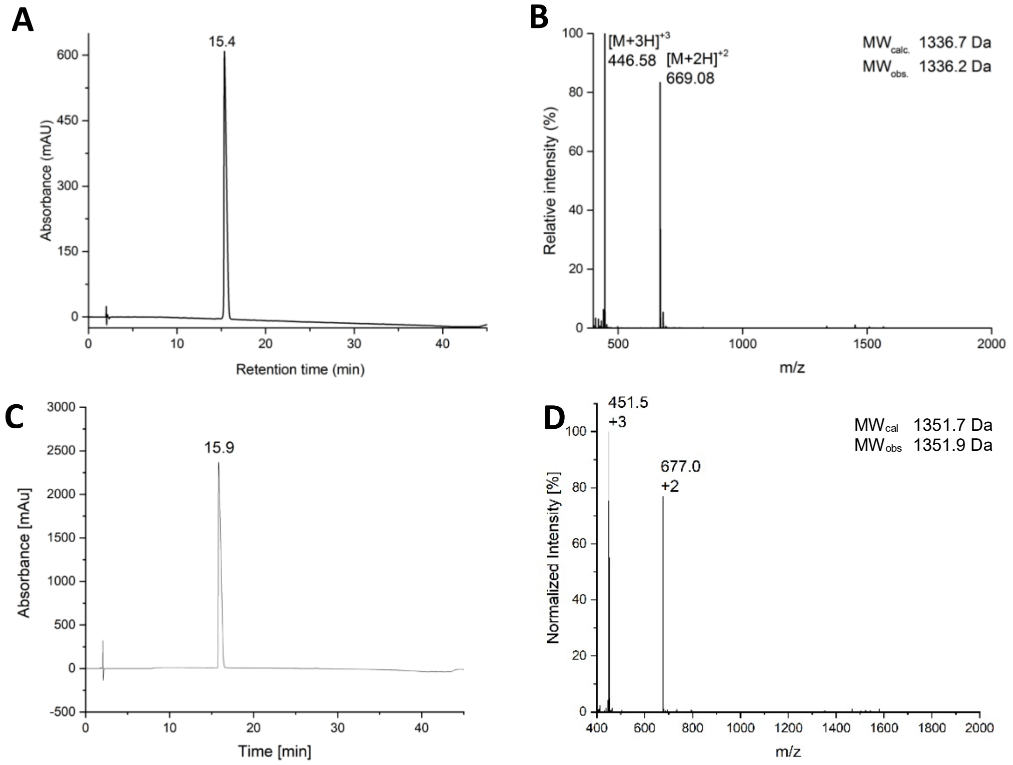

Figure S7: A: HPLC chromatogram of purified peptide **4**. B: Mass spectrum of purified peptide **4.** C: HPLC chromatogram of purified peptide **4’**. D: Mass spectrum of purified peptide **4’.**

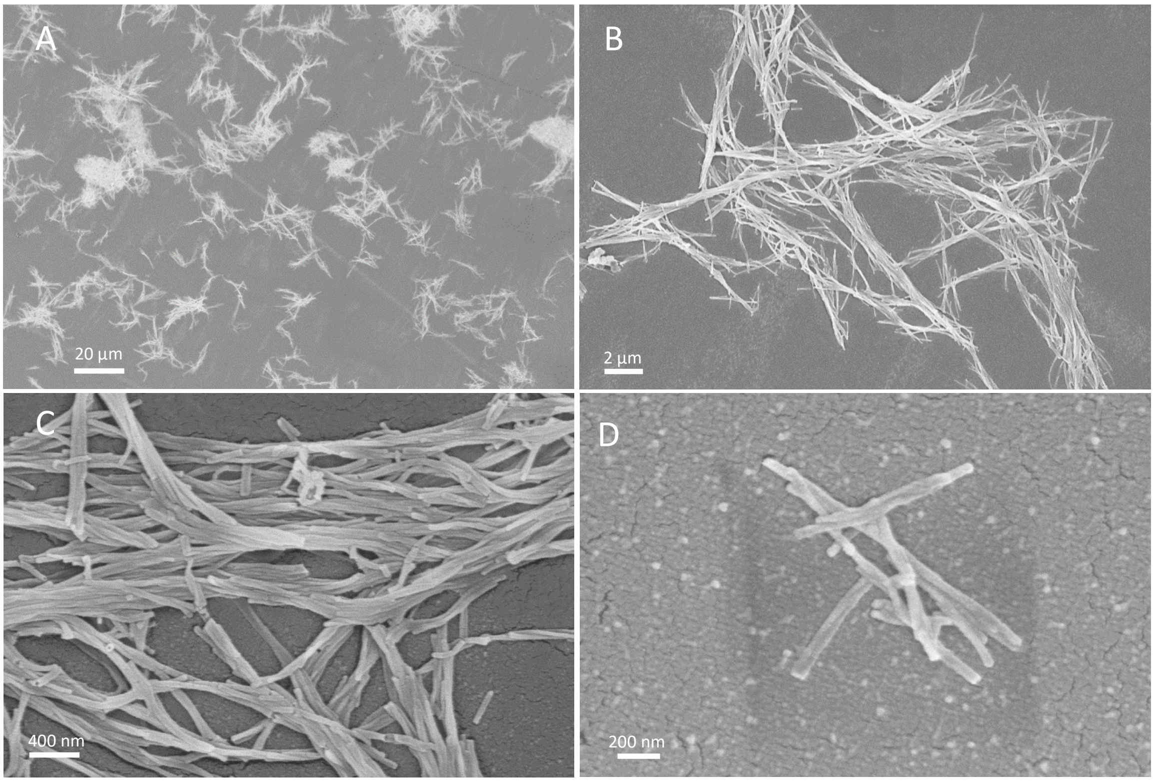

Figure S8a: A-D) Selection of different SEM images of particles obtained after silica precipitation with peptide **4**.

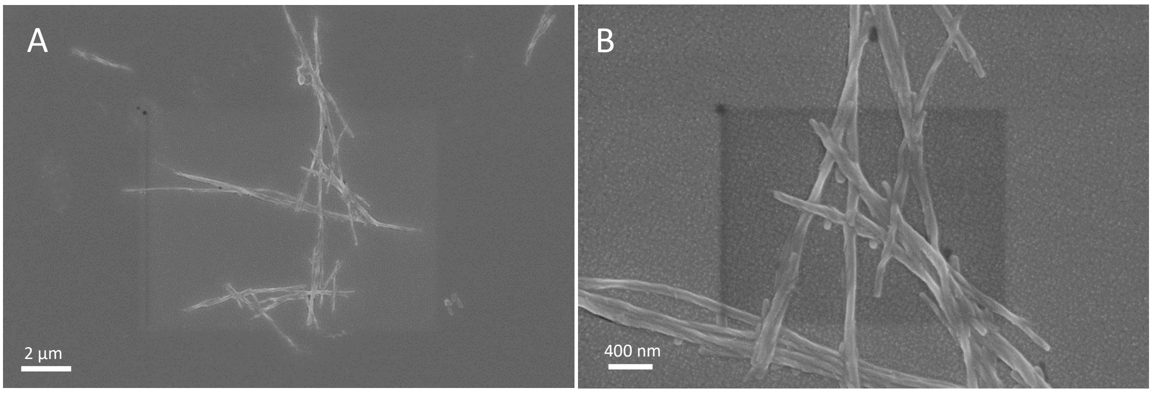

Figure S8b: A-B) Different SEM images of the precipitate obtained during phosphate buffer incubation with peptide **4**.

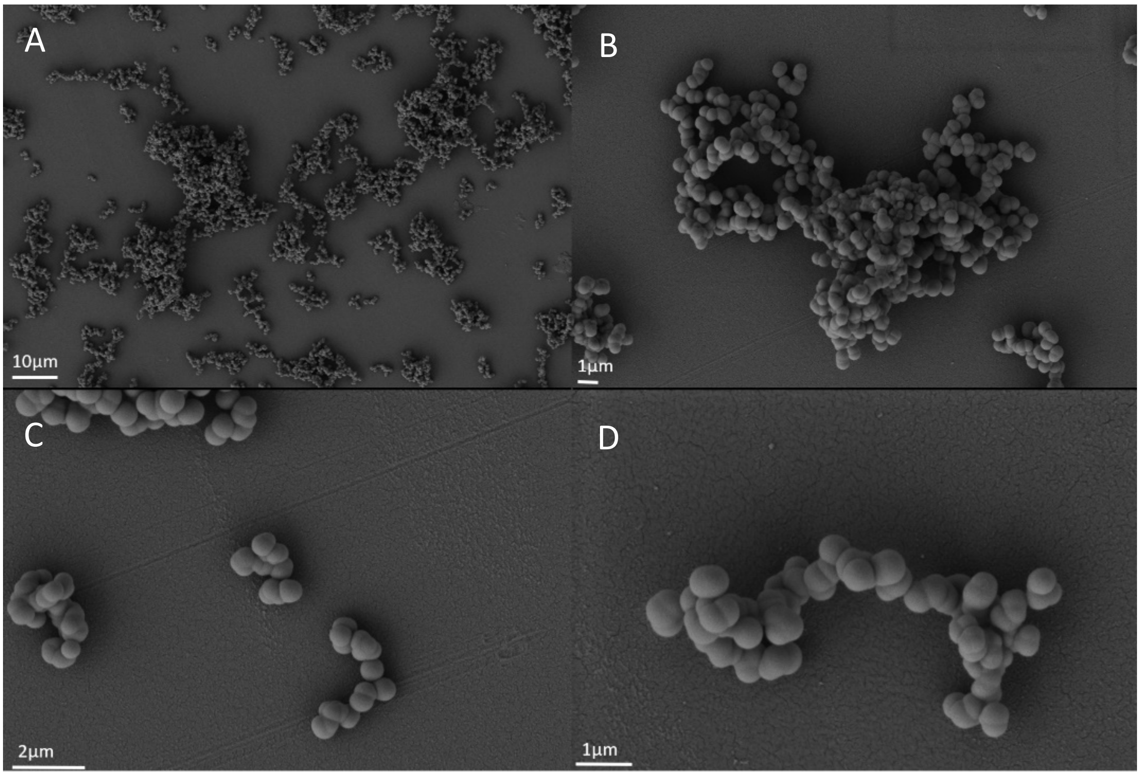

Figure S8c: A-D) Selection of different SEM images of particles obtained after silica precipitation with peptide **4'**.

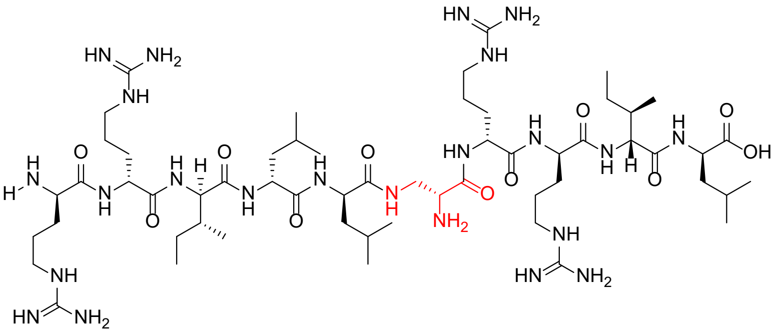

Scheme S5: Structure of peptide **5** with D-Dap as central residue. We obtained 7.9 mg (61 % for a 0.01 mmol synthesis scale).

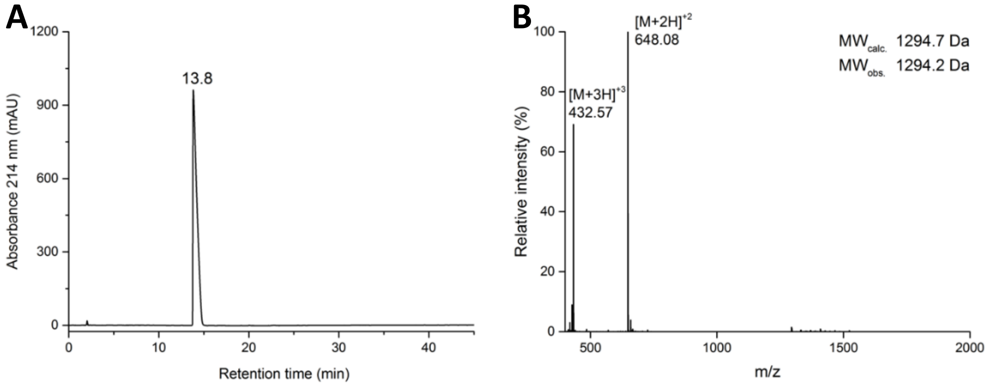

Figure S9: A: HPLC chromatogram of purified peptide **5**. B: Mass spectrum of purified peptide **5**.

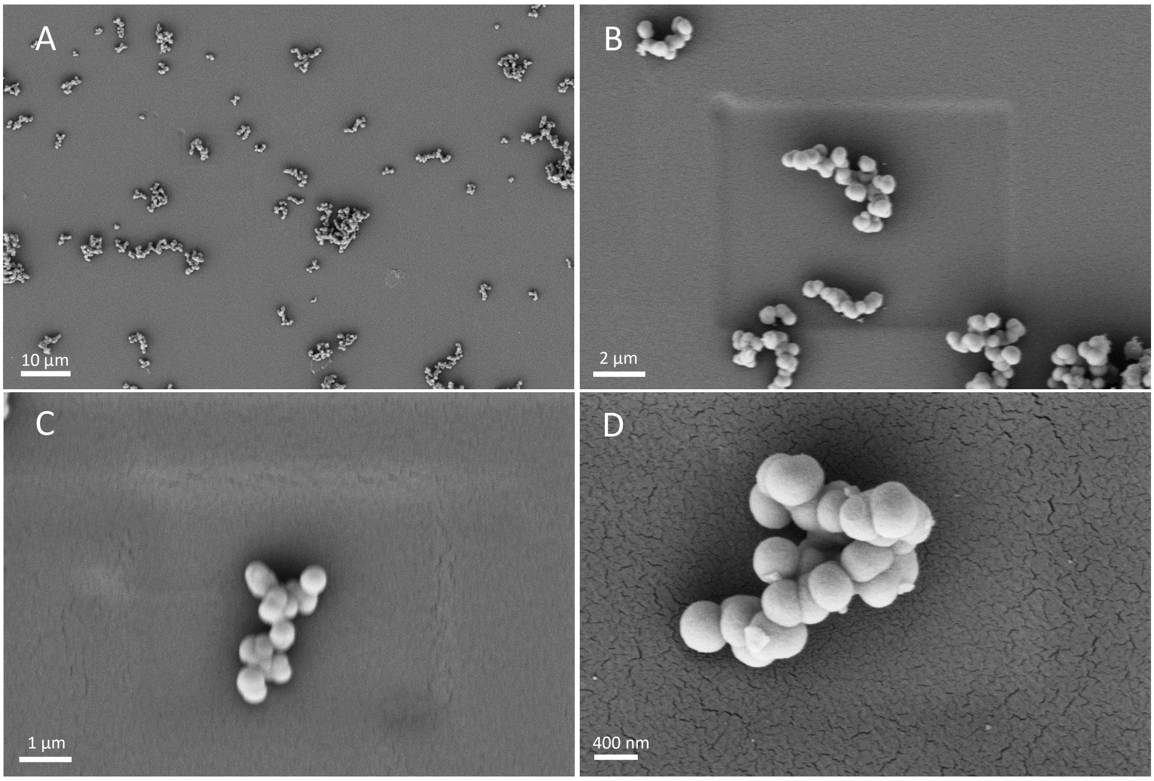

Figure S10: A-D) Selection of different SEM images of particles obtained after silica precipitation with peptide **5**.

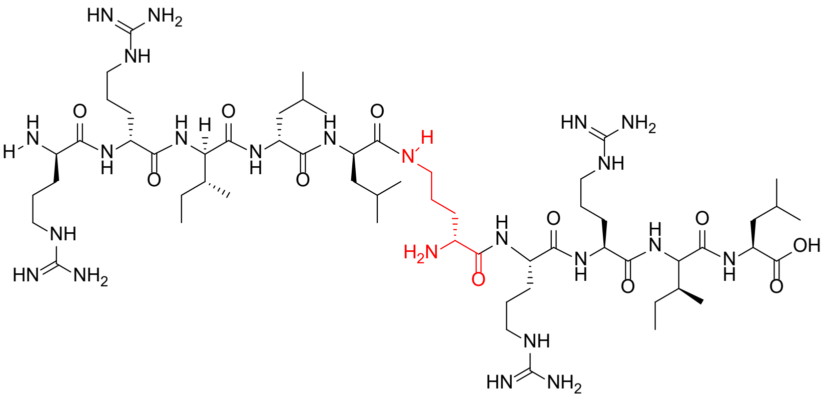

Scheme S6: Structure of peptide **6** with D-Ornithine as a central residue. We obtained 8.4 mg (64 % for a 0.01 mmol synthesis scale).

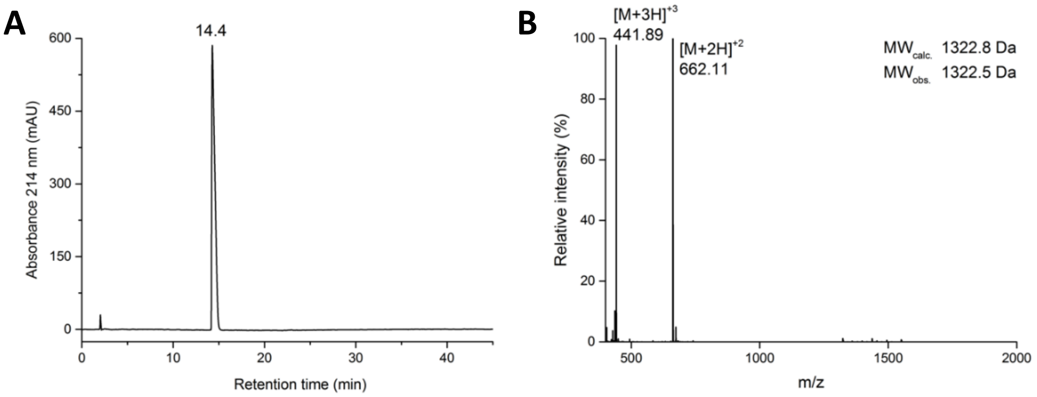

Figure S11: A: HPLC chromatogram of purified peptide **6**. B: Mass spectrum of purified peptide **6**.

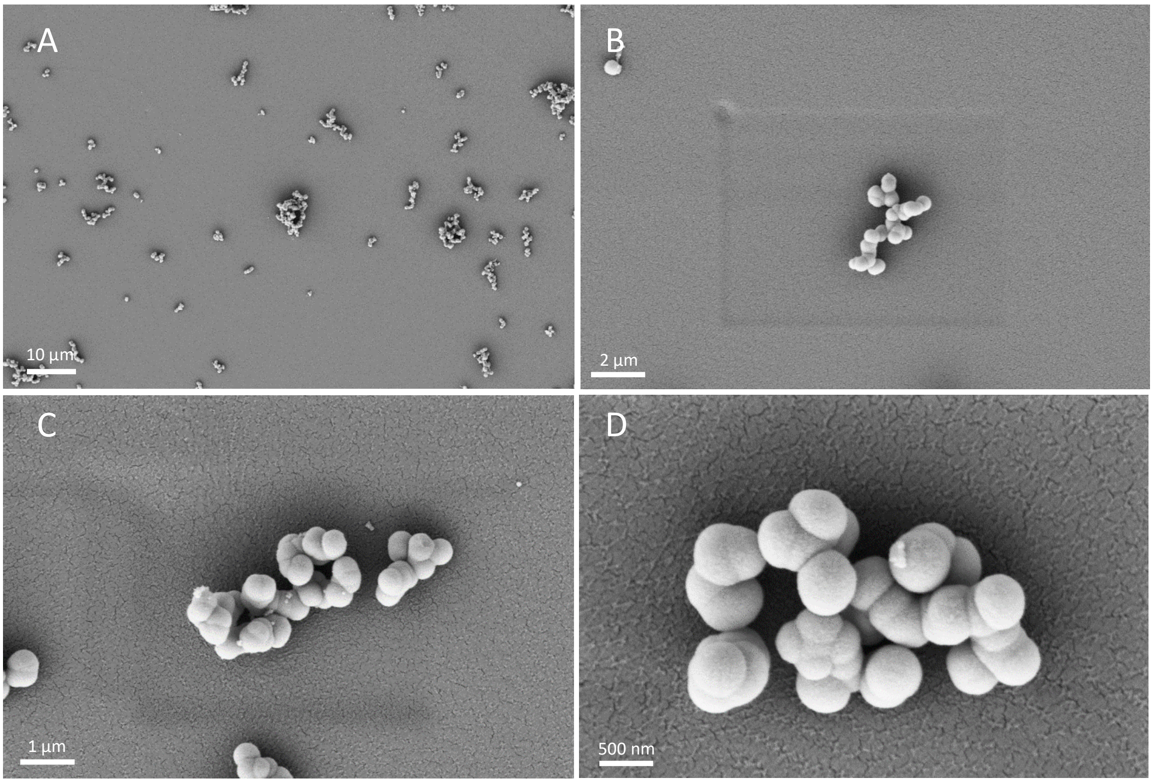

Figure S12: A-D) Selection of different SEM images of particles obtained after silica precipitation with peptide **6**.

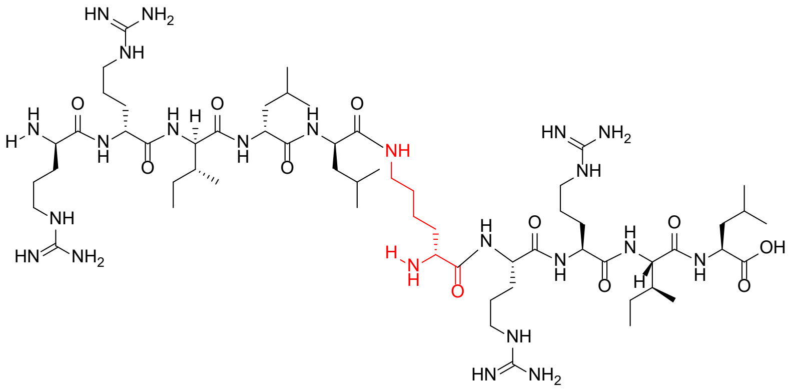

Scheme S7: Structure of peptide **7** with D-Lysine as a central residue. We obtained 12.3 mg (92 % for a 0.01 mmol synthesis scale).

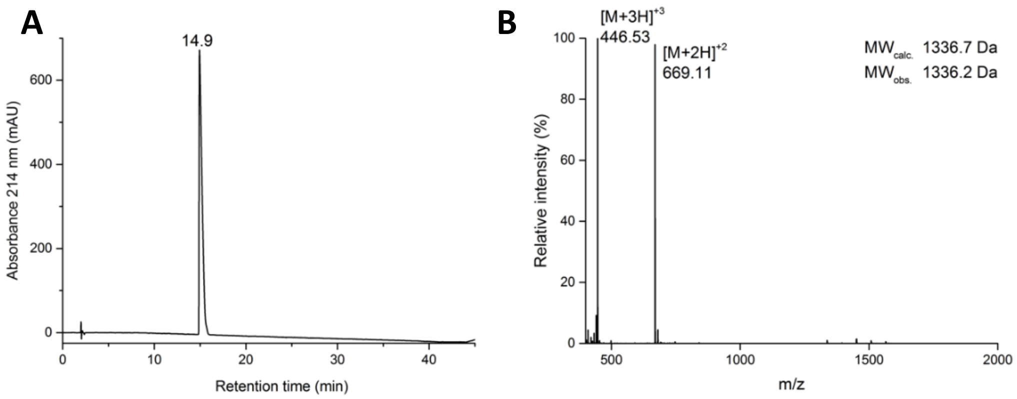

Figure S13: A: HPLC chromatogram of purified peptide **7**. B: Mass spectrum of purified peptide **7**.

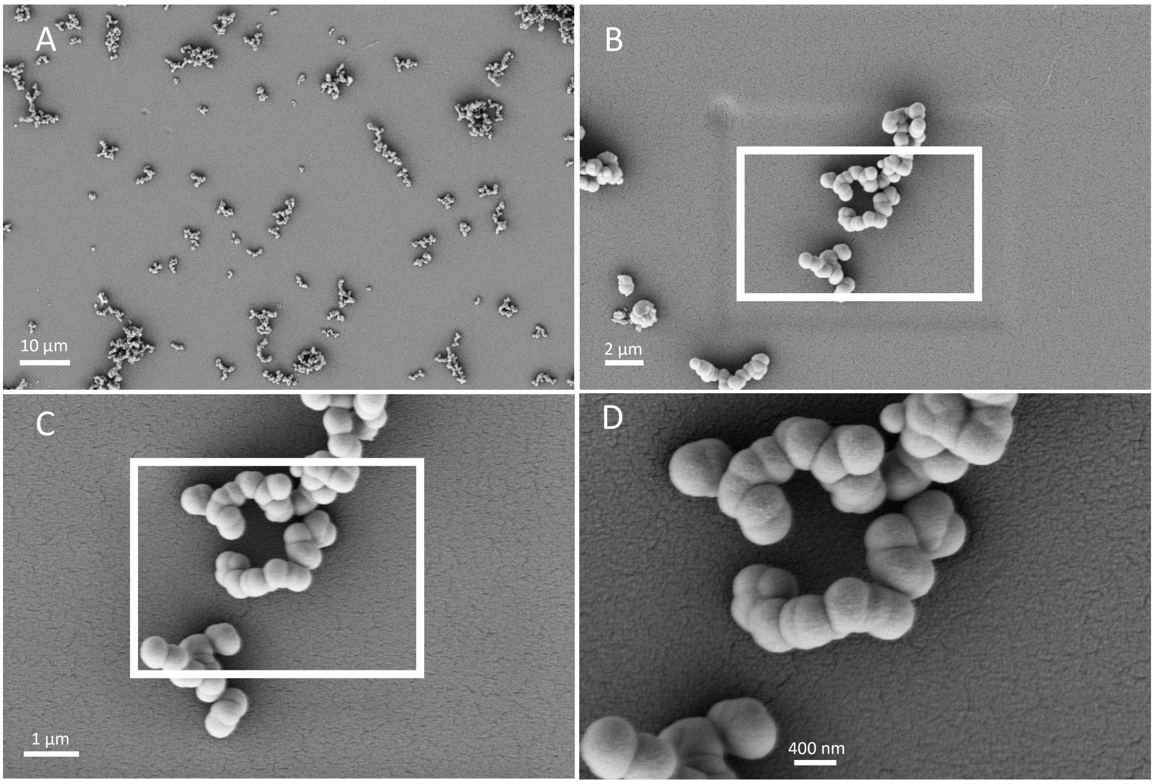

Figure S14: A) SEM image of particles obtained after silica precipitation peptide **7**. B) Zoom-in to the marked area in B). C) Zoom-in to the marked area in panel B). D) Zoom- in to the marked area in panel C).

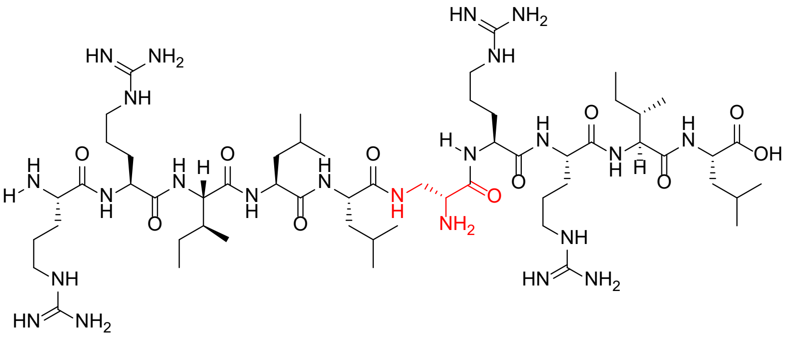

Scheme S8: Structure of peptide **8** with D-Dap as central residue. We obtained 10.5 mg (81 % for a 0.01 mmol synthesis scale).

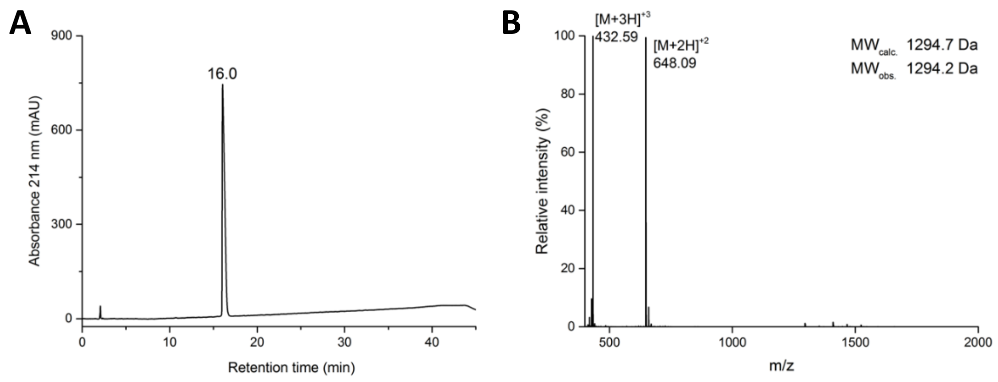

Figure S15: A: HPLC chromatogram of purified peptide **8**. B: Mass spectrum of purified peptide **8**.

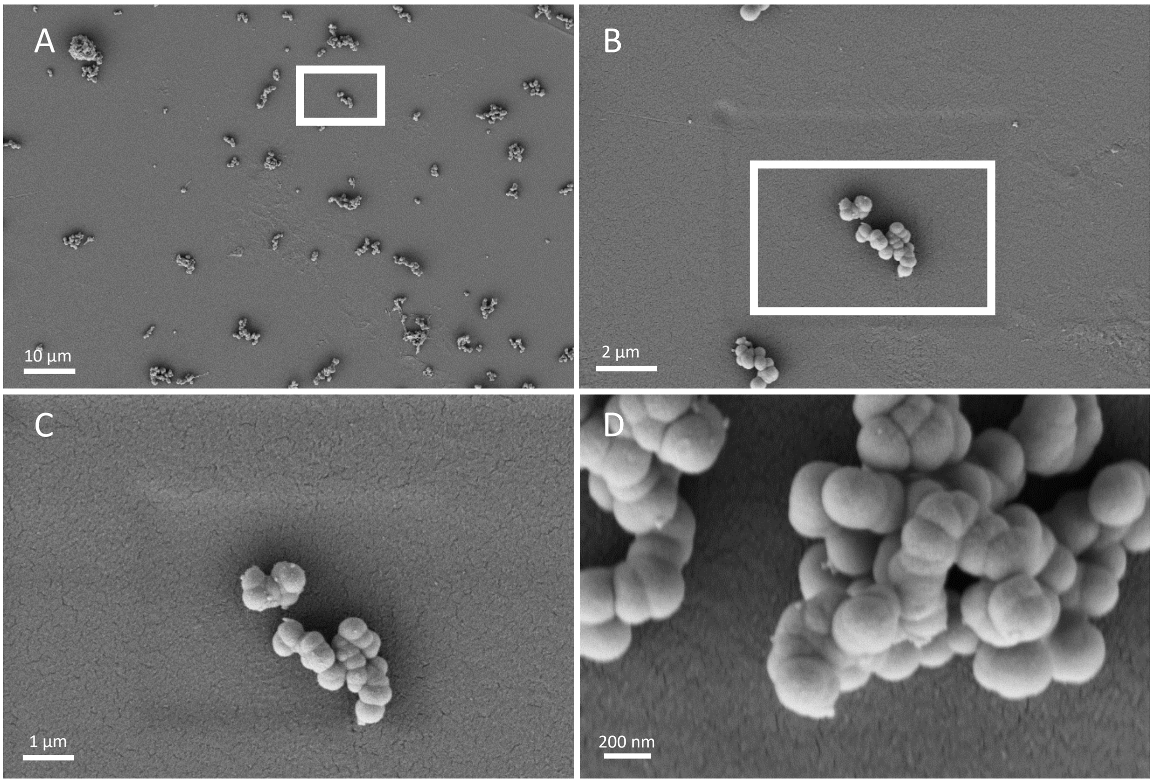

Figure S16: A) SEM image of particles obtained after silica precipitation with peptide **8**. B) Zoom-in to the marked area in panel A). C) Zoom-in to the marked area in panel B). D) SEM image of particles obtained after silica precipitation with peptide **8** in different magnification.

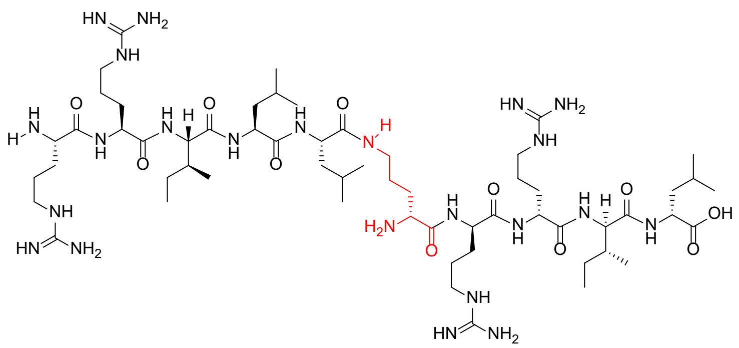

Scheme S9: Structure of peptide **9** with D-Ornithine as a central residue. We obtained 10.1 mg (77 % for a 0.01 mmol synthesis scale).

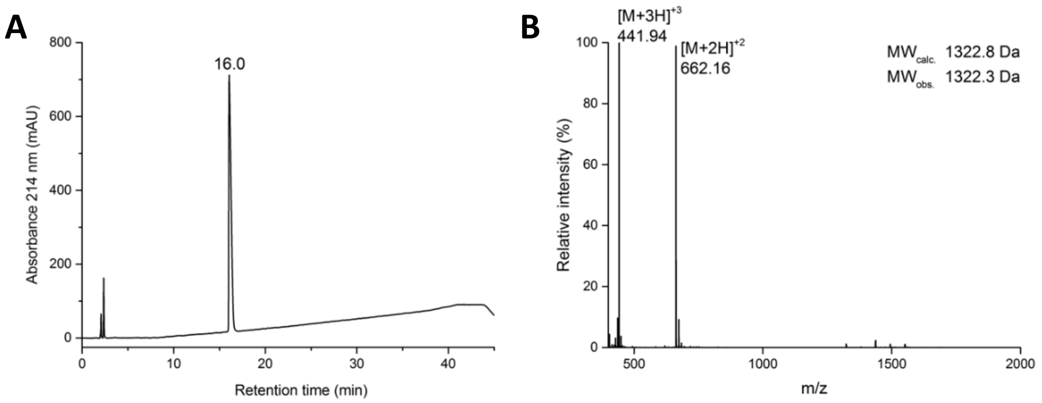

Figure S17: A: HPLC chromatogram of purified peptide **9**. B: Mass spectrum of purified peptide **9**.

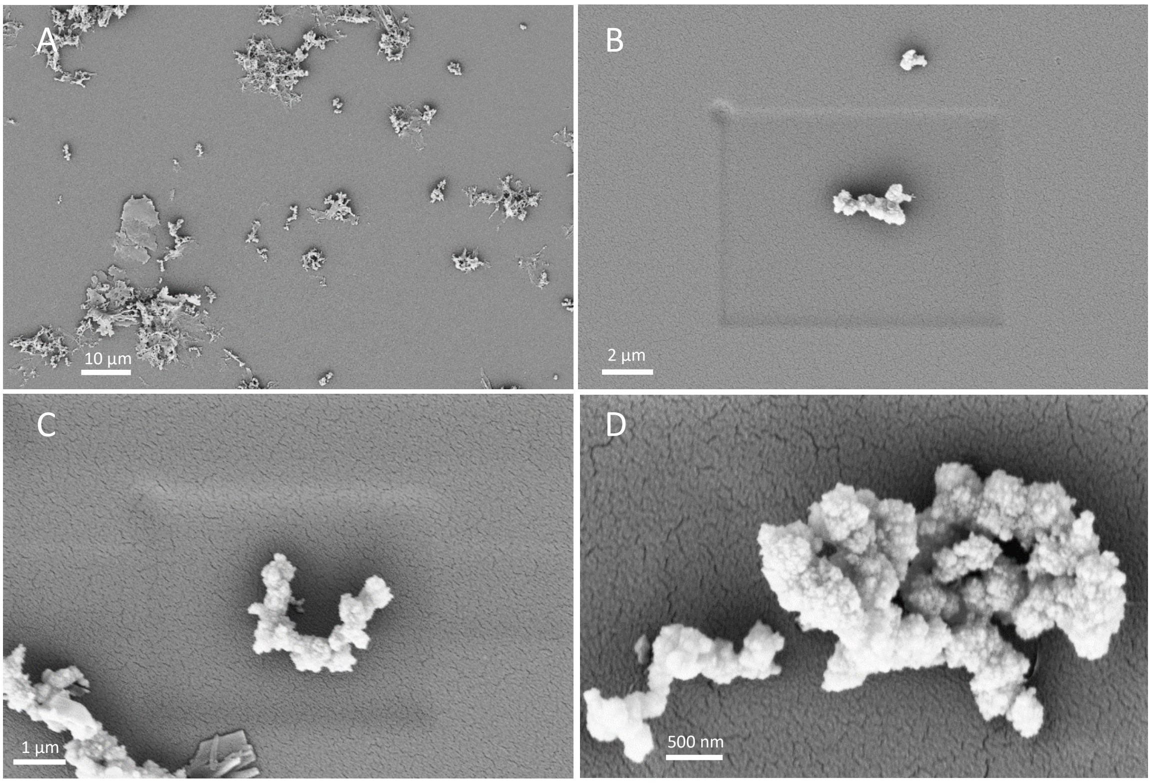

Figure S18a: A-D) Selection of different SEM images of particles obtained after silica precipitation with peptide **9**.

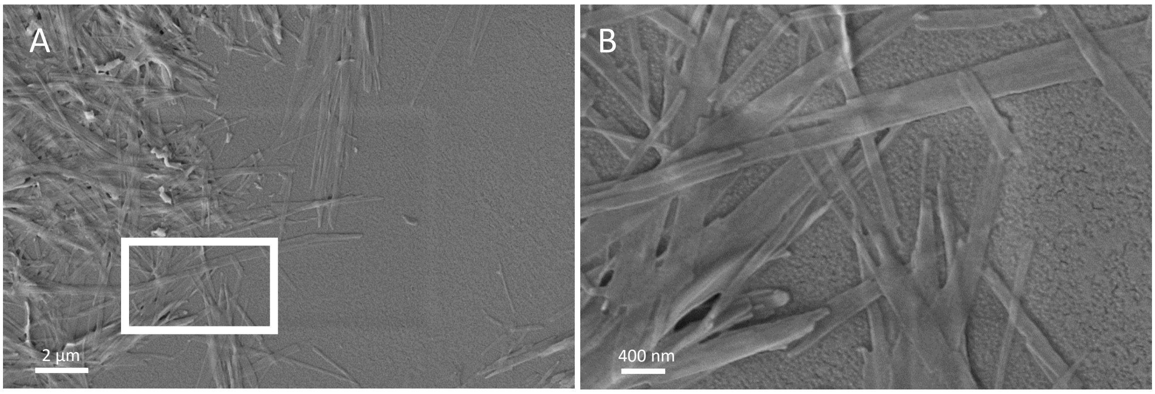

Figure S18b: A) SEM image of peptide **9** precipitate obtained during incubation with phosphate buffer. B) Zoom-in to the marked area of panel A).

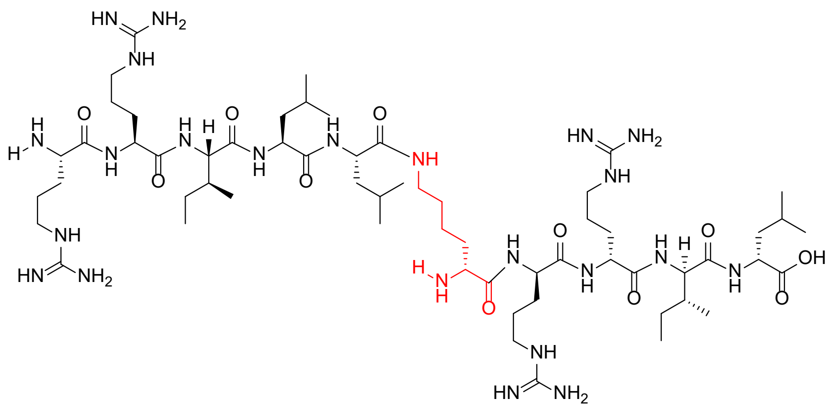

Scheme S10: Structure of peptide **10** with D-Lysine as a central residue. We obtained 8.2 mg (62 % for a 0.01 mmol synthesis scale).

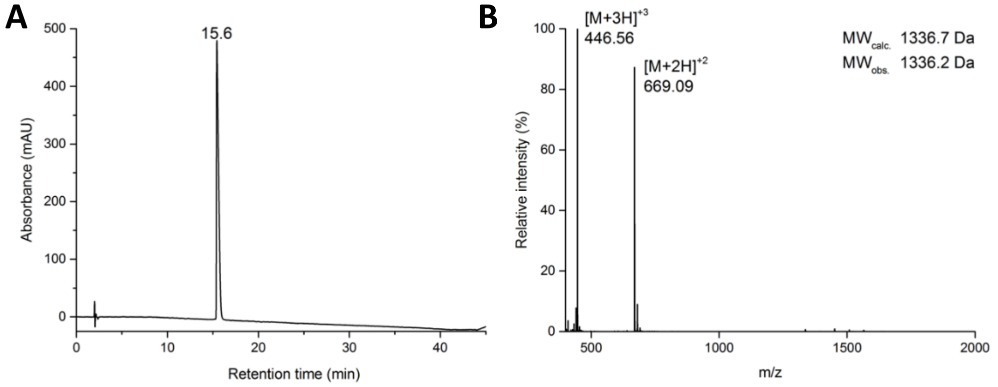

Figure S19: A: HPLC chromatogram of purified peptide **10**. B: Mass spectrum of purified peptide **10**.

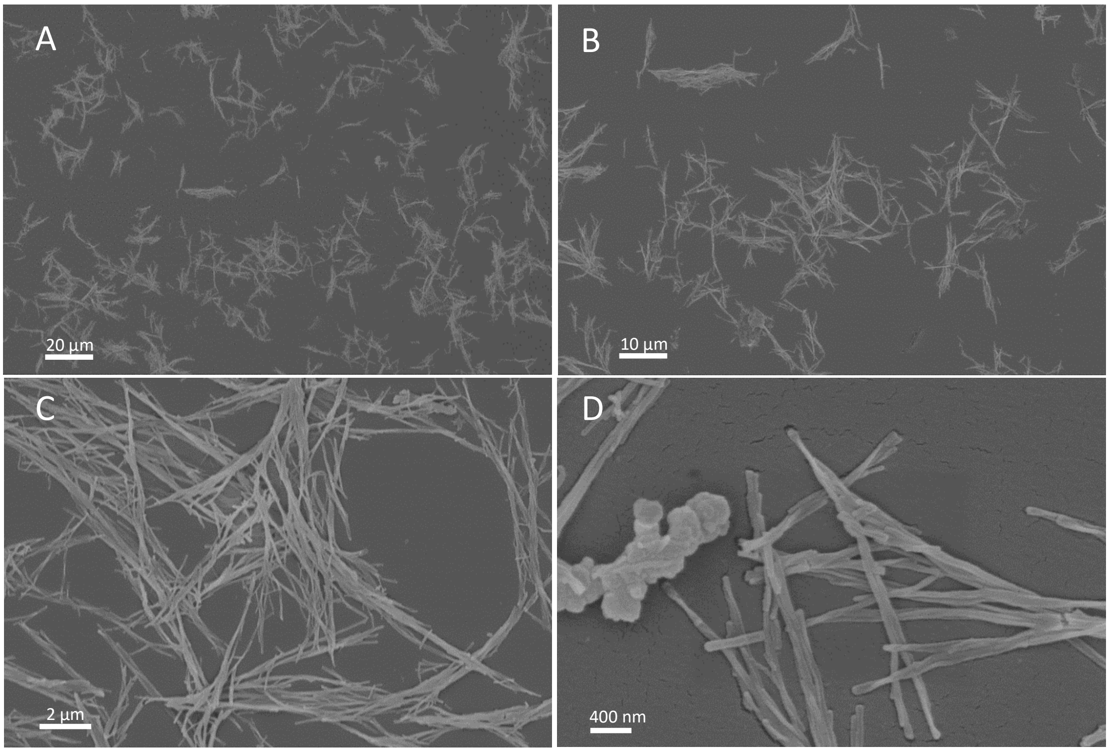

Figure S20a: A-D) Selection of different SEM images of particles obtained after silica precipitation with peptide **10**.

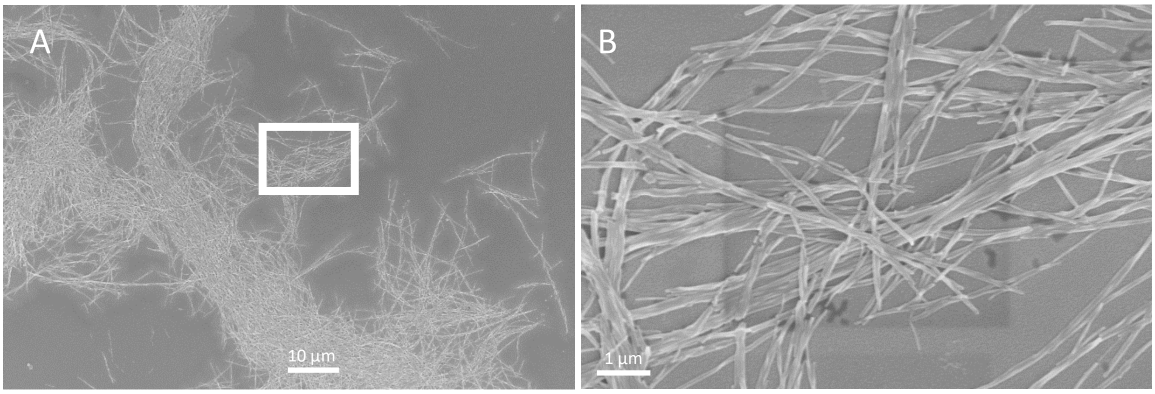

Figure S20b: A) SEM image of peptide **10** precipitate obtained during incubation with phosphate buffer. B) Zoom-in on the marked area of panel A).

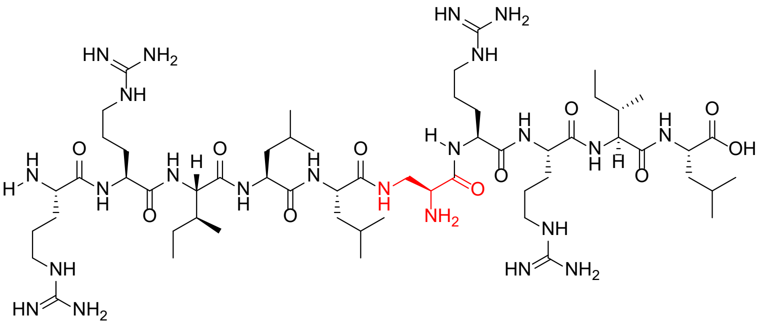

Scheme S11: Structure of peptide **11** with L-Dap as central residue. We obtained 9.0 mg (70 % for a 0.01 mmol synthesis scale).

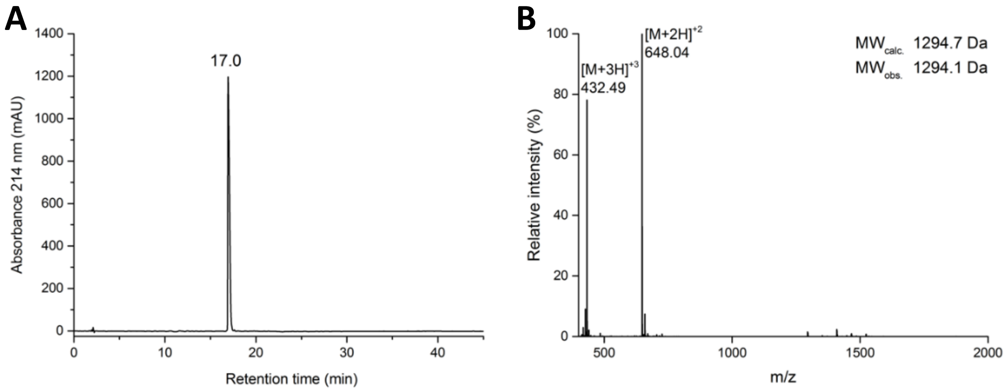

Figure S21: A: HPLC chromatogram of purified peptide **11**. B: Mass spectrum of purified peptide **11**.

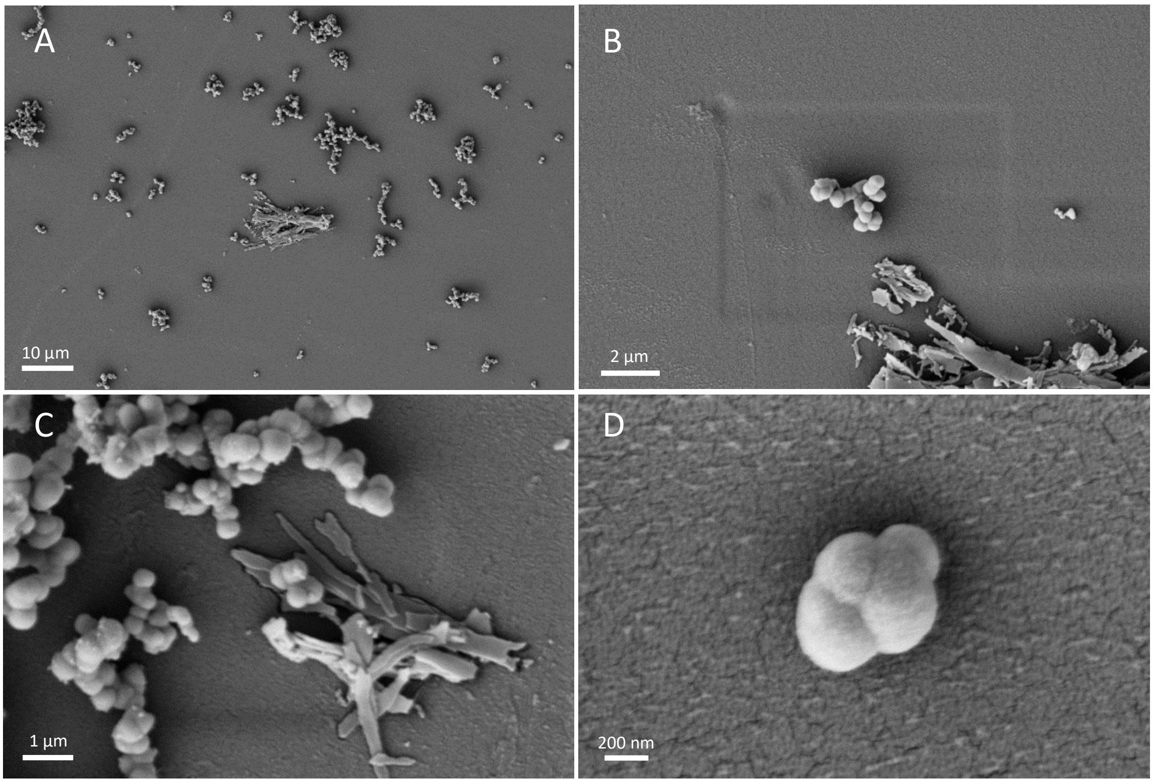

Figure S22: A-D) Selection of SEM images of particles obtained after silica precipitation with peptide **11**.

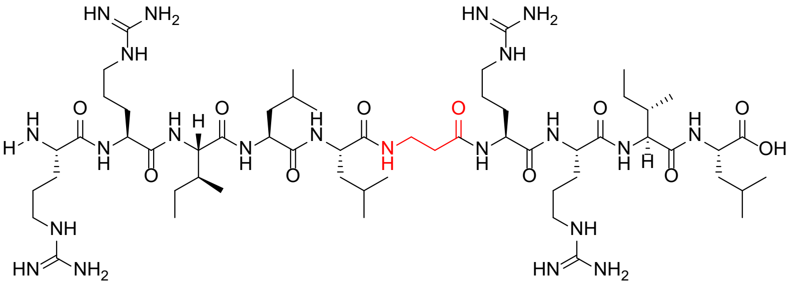

Scheme S12: Structure of peptide **12** with β-Alanine as a central residue. We obtained 4.9 mg (38 % for a 0.01 mmol synthesis scale).

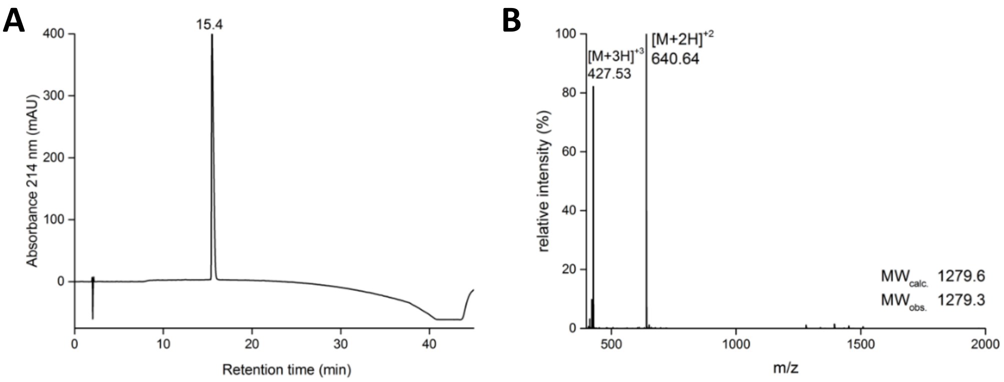

Figure S23: A: HPLC chromatogram of purified peptide **12**. B: Mass spectrum of purified peptide **12**.

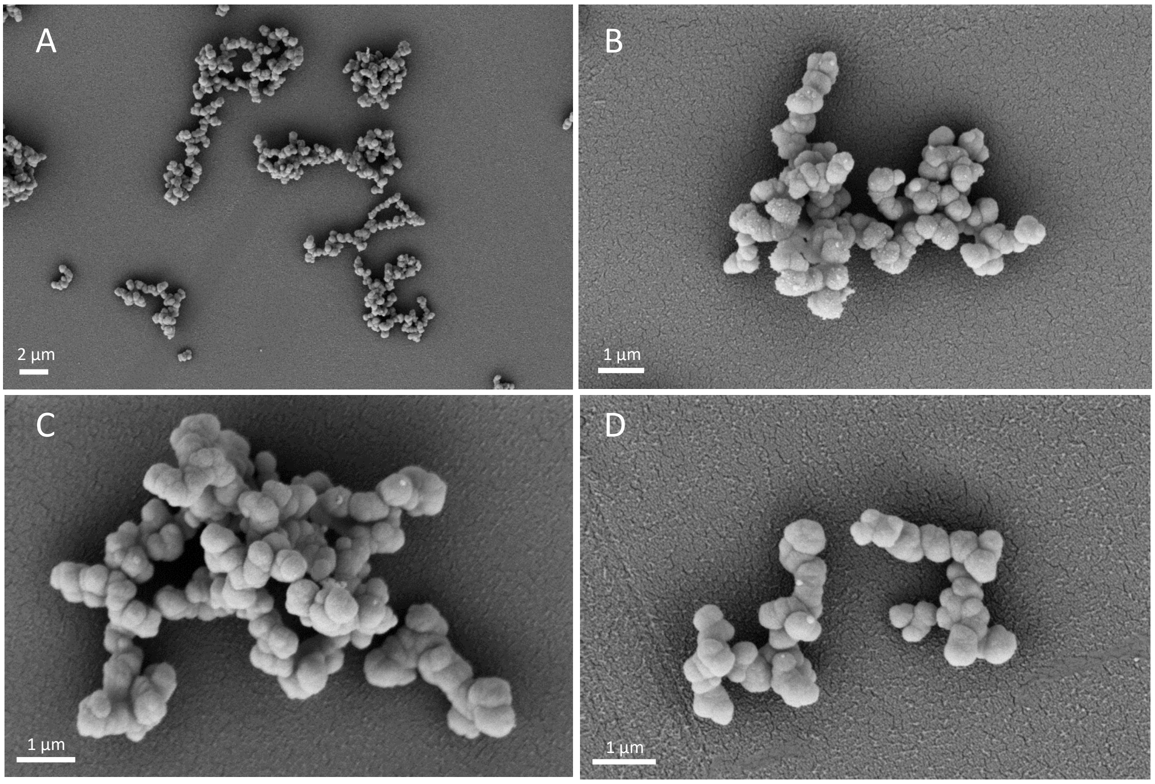

Figure S24: A-D) Selection of SEM images of particles obtained after silica precipitation with peptide **12**.

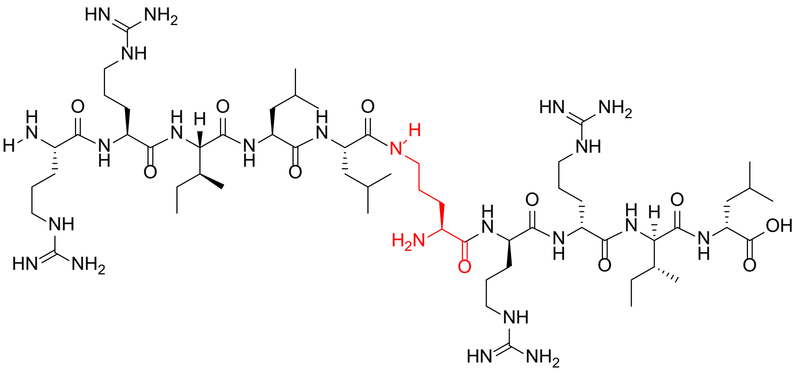

Scheme S13: Structure of peptide 13 with L-Ornithine as a central residue. We obtained 8.9 mg (67 % for a 0.01 mmol synthesis scale).

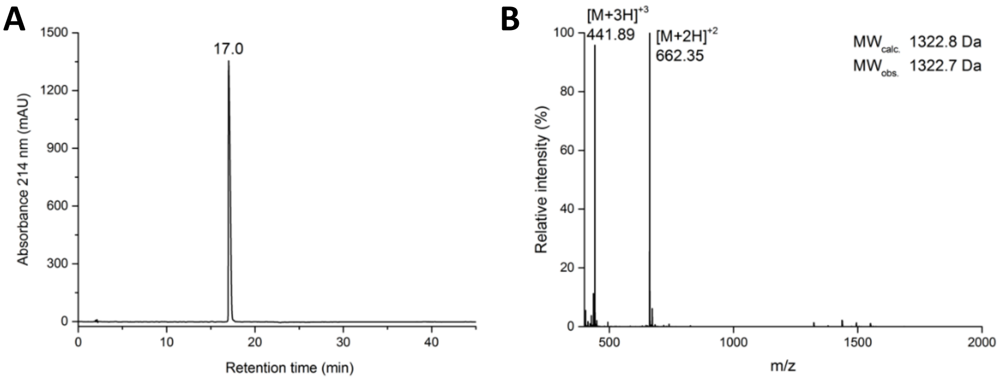

Figure S25: A: HPLC chromatogram of purified peptide **13**. B: Mass spectrum of purified peptide **13**.

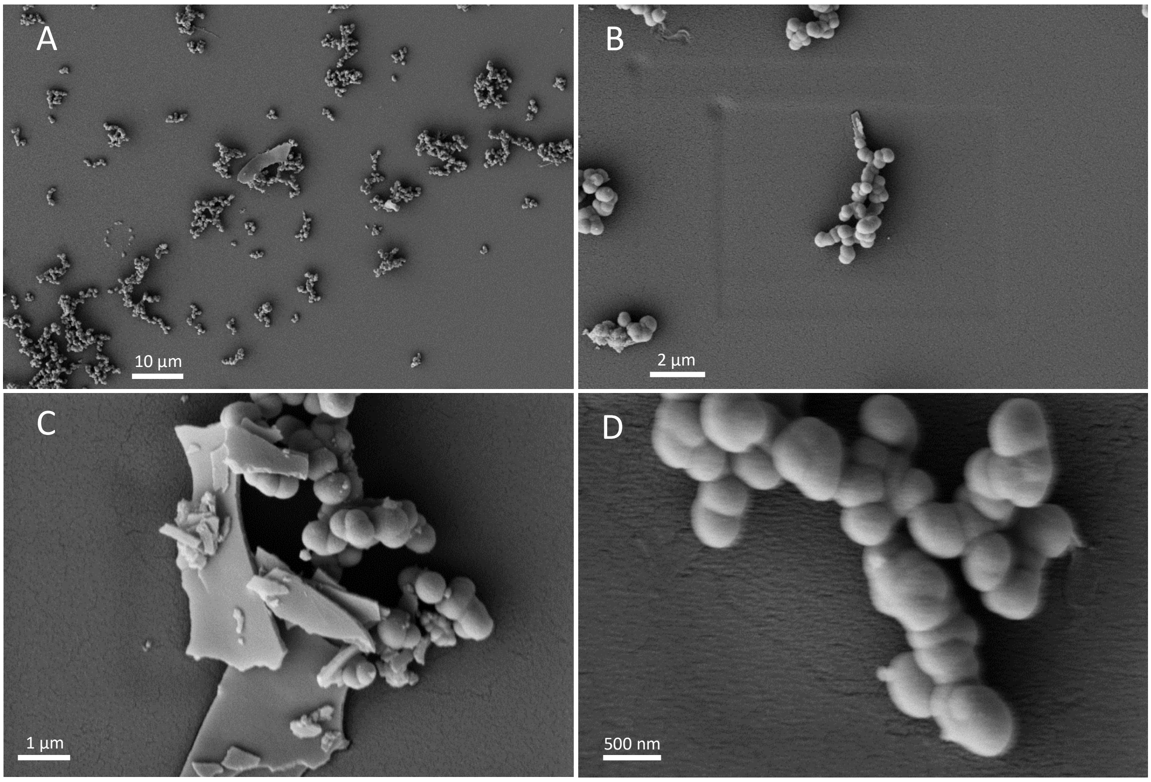

Figure S26: A-D) Selection of SEM images of particles obtained after silica precipitation with peptide **13**.

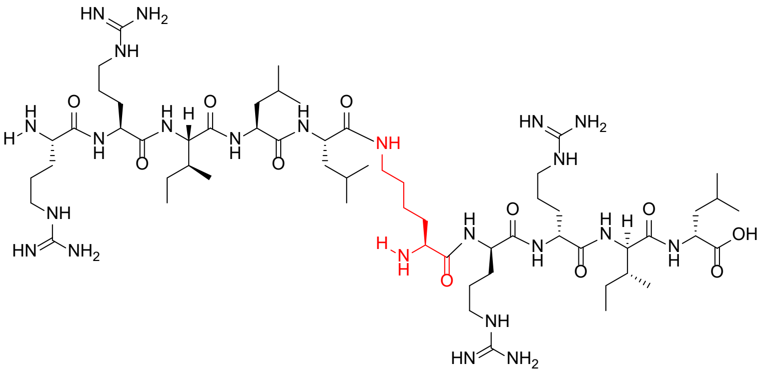

Scheme S14: Structure of peptide **14** with L-Lysine as a central residue. We obtained 5.2 mg (39 % for a 0.01 mmol synthesis scale).

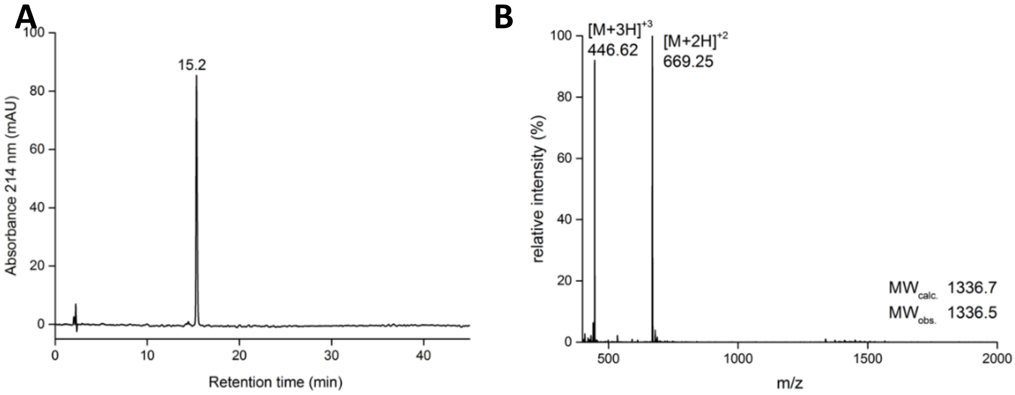

Figure S27: A: HPLC chromatogram of purified peptide **14**. B: Mass spectrum of purified peptide **14**.

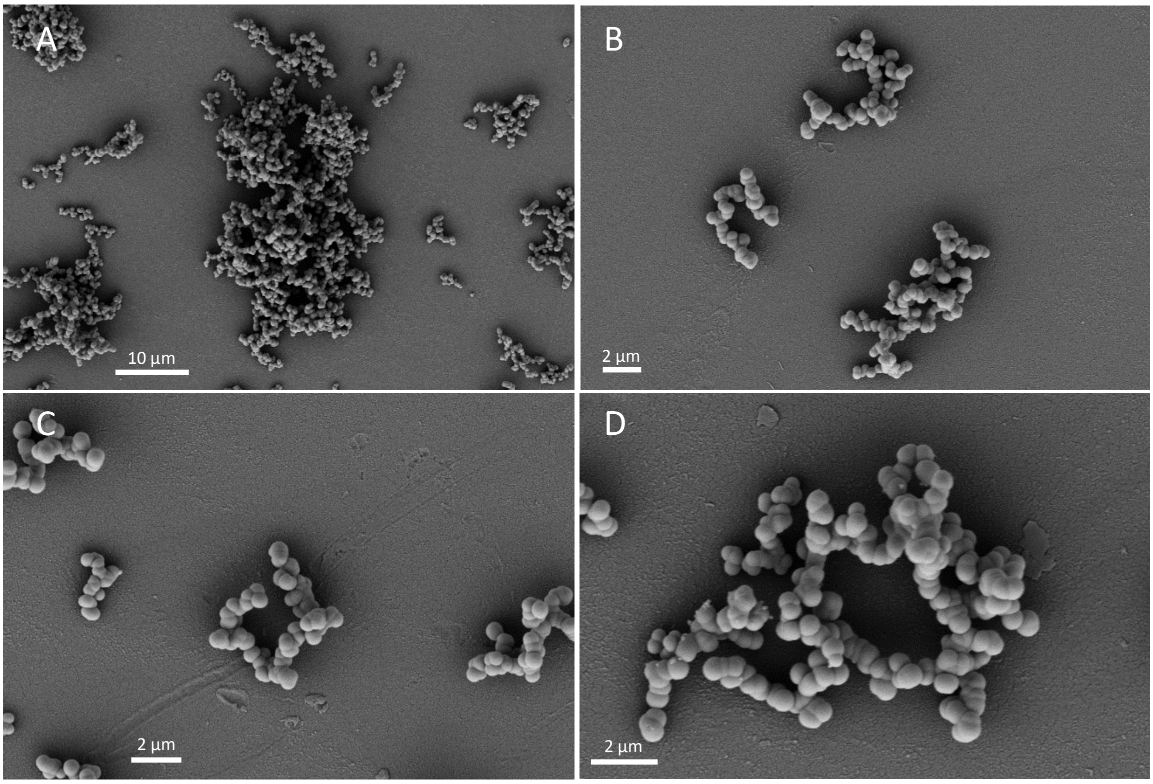

Figure S28: A-D) Selection of SEM images of particles obtained after silica precipitation with peptide **14**.

Supplementary MD & NMR data

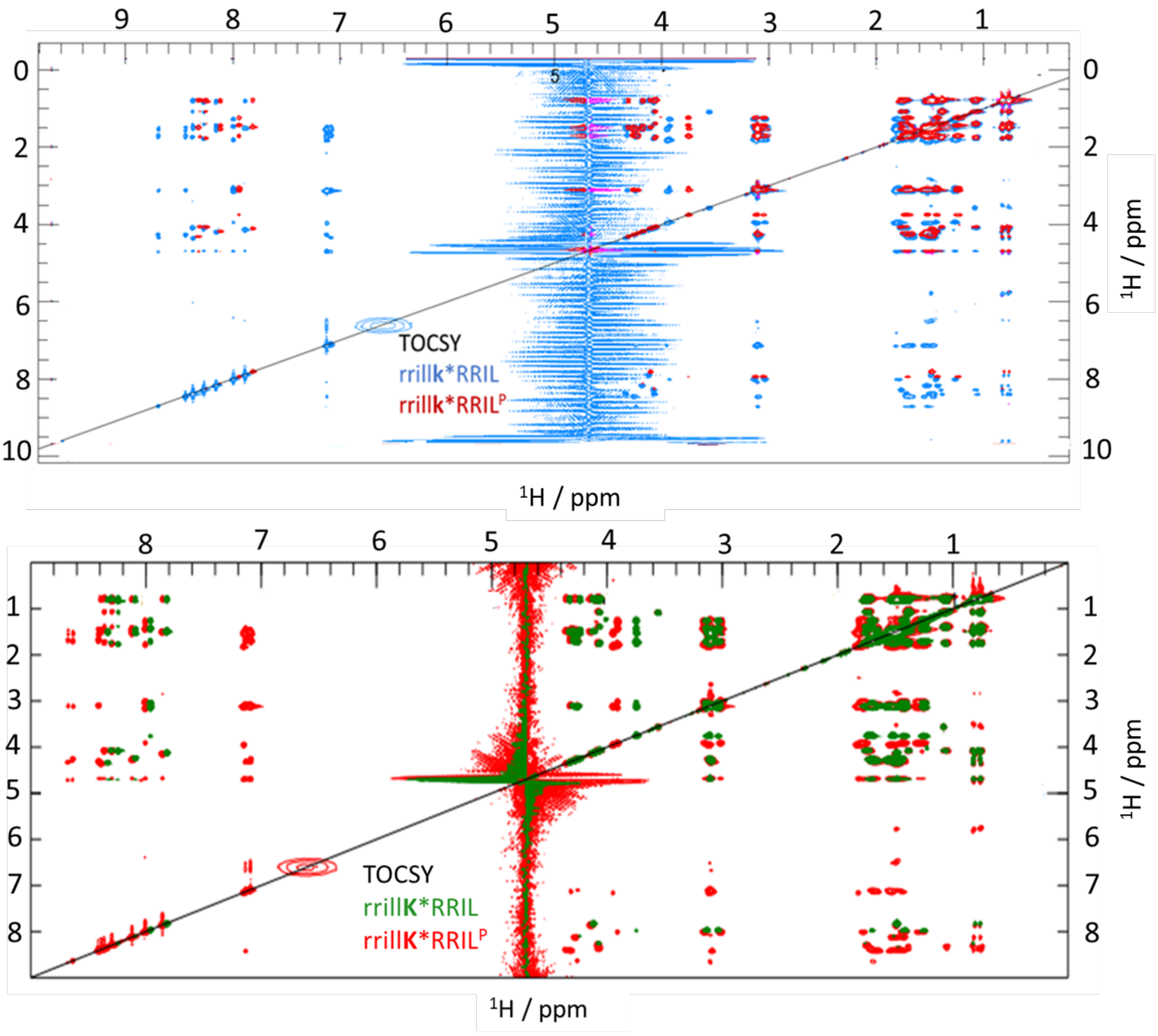

Figure S29. Supplementary full TOCSY spectra of rrillK*RRIL **4** and rrillk*RRIL **7** in water (blue and green) and 50 mM PBS (red and green).

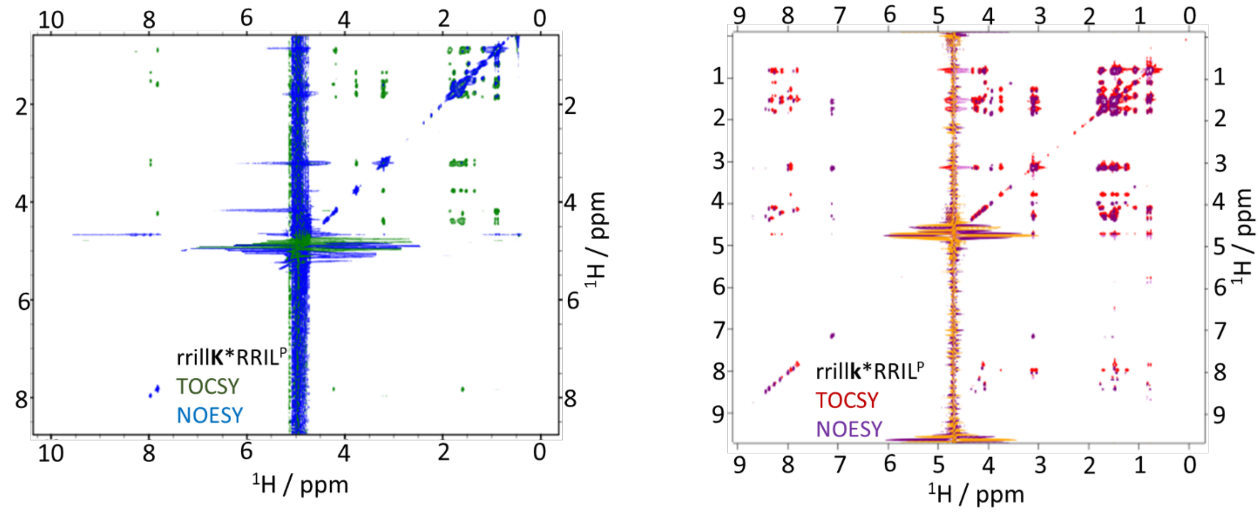

Figure S30. NOESY and TOCSY spectra overlay of peptides rrillK*RRIL **4** (left, blue and green respectivley) and rrillk*RRIL **7** (right, red and purple respectively).

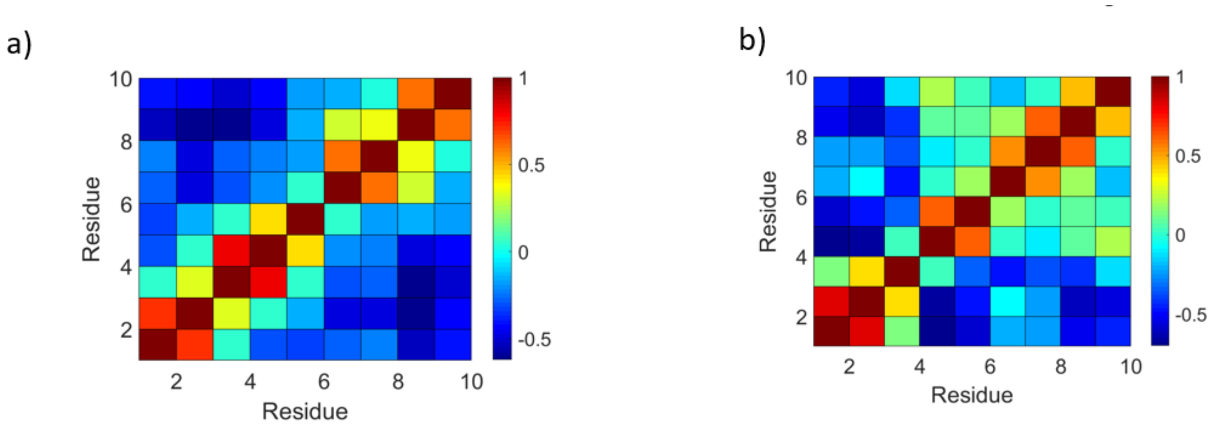

Figure S31. a) Dynamic cross-correlation (DCC) matrix for rrillk*RRIL in PBS. A high DCC between two residues indicates correlated motion and structural proximity (*e.g.*, hydrophobic contact or a hydrogen bond). No long-range correlations can be observed. b) Dynamic cross-correlation (DCC) matrix for rrillK*RRIL. In contrast to panel a, residues K^{6} to R^{10} form a compacted structure, which also correlates with residues r^{1} to l^{5}.

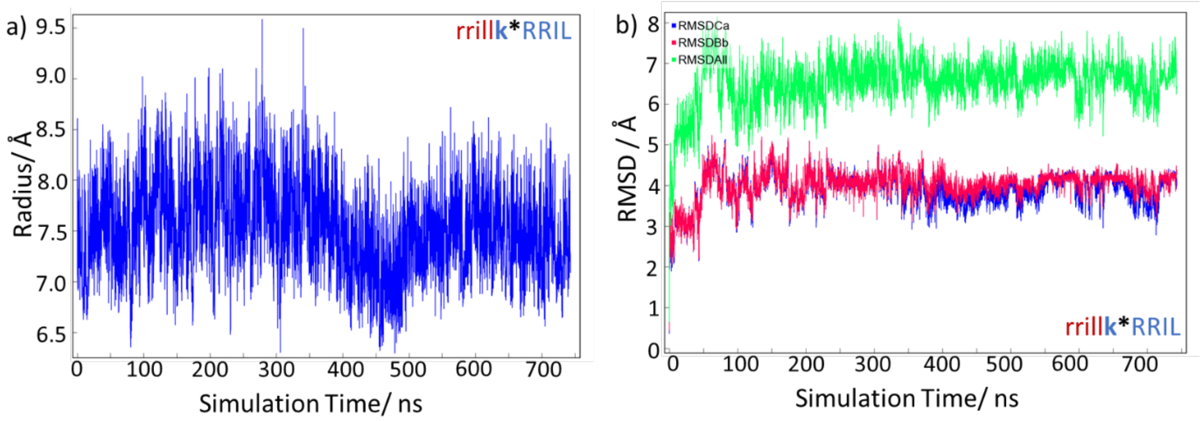

Figure S32. a) Radius of Gyration for rrillk*RRIL **7** over 700ns trajectory in PBS as calculated by $Radius_{gyr,Mass} = \sqrt{\frac{\sum_{i=1}^{N} Mass_i(\vec{R_i}-\vec{C})^2}{\sum_{i=1}^{N} Mass_i}}$.[1] b) RMSD of rrillk*RRIL **7** over 700ns trajectory in PBS as calculated by $RMSD = \sqrt{\frac{\sum_{i=1}^{n} R_i * R_i}{n}}$, with R_i as the vector between reference and snapshot at a given simulation time.[2]

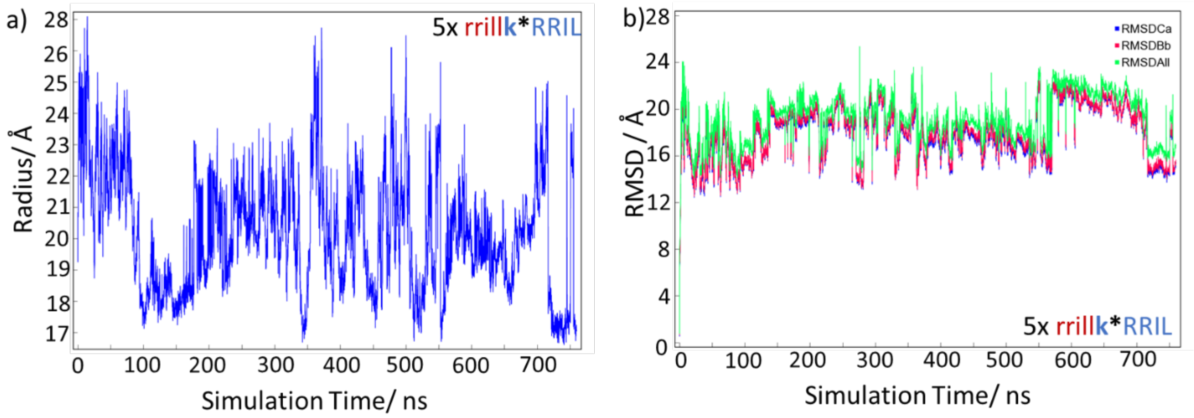

Figure S33. a) Radius of Gyration for 5 units rrillk*RRIL **7** over 700ns trajectory in PBS as calculated in S32. b) RMSD of 5 units rrillk*RRIL **7** over 700ns trajectory in PBS as calculated in S32.

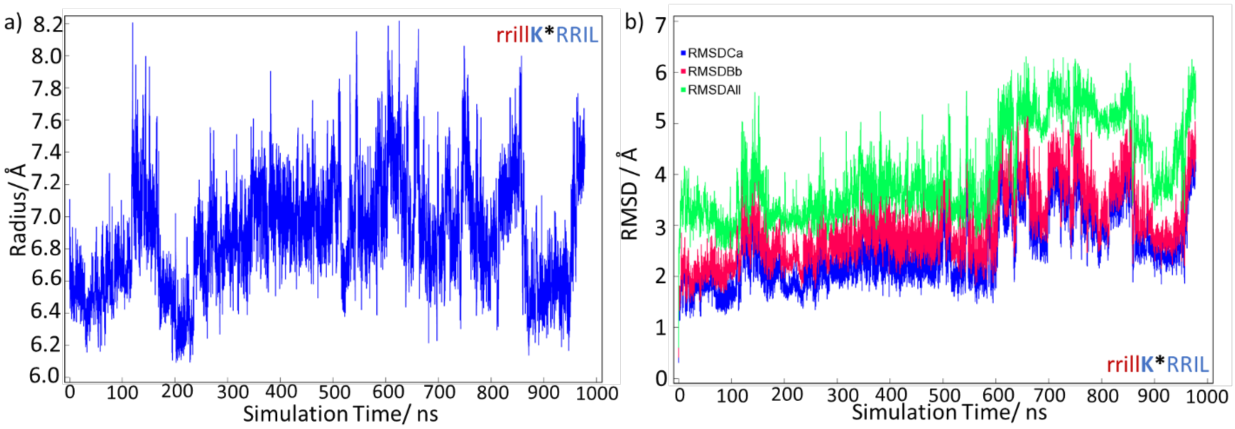

Figure S34. a) Radius of Gyration for rrillK*RRIL **4** over 700ns trajectory in PBS as calculated by $Radius_{gyr,Mass} = \sqrt{\frac{\sum_{i=1}^{N} Mass_i(\vec{R_i}-\vec{C})^2}{\sum_{i=1}^{N} Mass_i}}$.[1] b) RMSD of rrillK*RRIL **4** over 700ns trajectory in PBS as calculated by $RMSD = \sqrt{\frac{\sum_{i=1}^{n} R_i * R_i}{n}}$, with R_i as the vector between reference and snapshot at a given simulation time.[2]

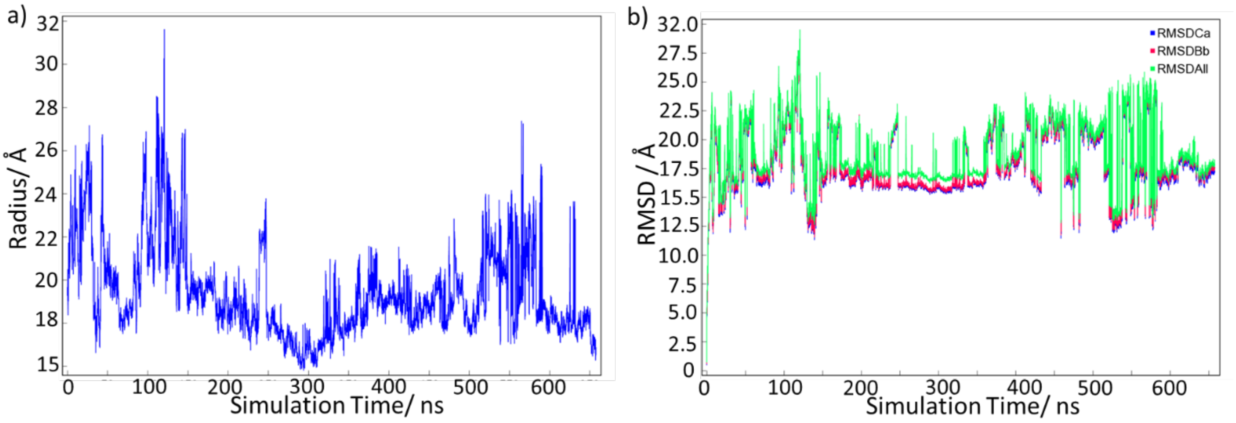

Figure S35. a) Radius of Gyration for 5 units rrillk*RRIL **4** over 660ns trajectory in PBS as calculated in S32. b) RMSD of 5 units rrillk*RRIL 4 over 700ns trajectory in PBS as calculated in S32.

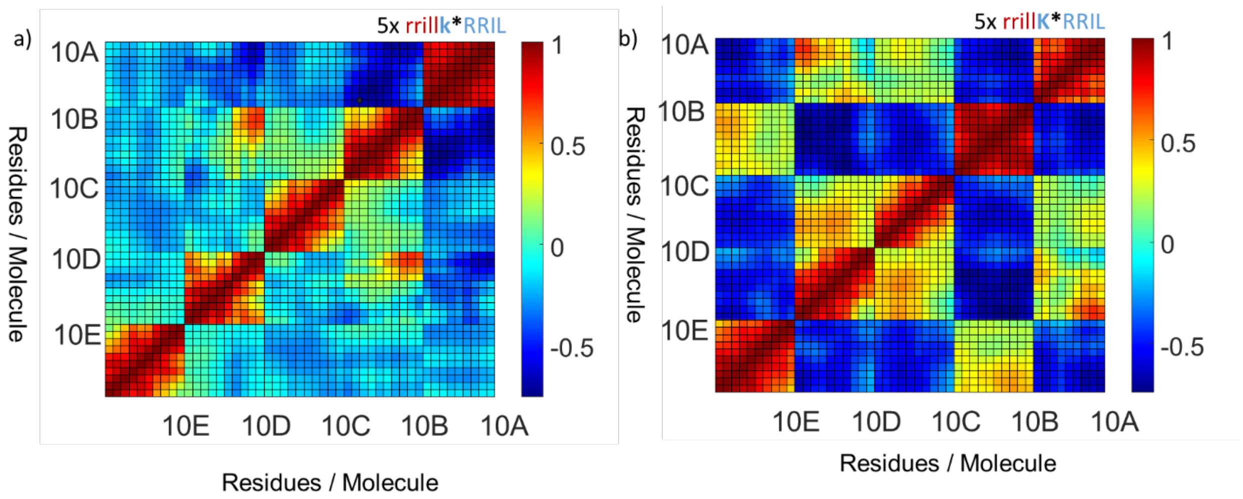

Figure S36. a) Dynamic cross correlation matrix of 5 units rrillk*RRIL **7** in PBS over 660ns. A high DCC between two residues indicates correlated motion and structural proximity. b) Dynamic cross correlation matrix of 5 units rrillK*RRIL **4** in PBS. As can be observed in comparison to a) higher density is shown by **4** mainly due to hydrophobic side-chain contacts. The DCCMs show that the features found for the monomers is retained in the complexes (Fig. S31).

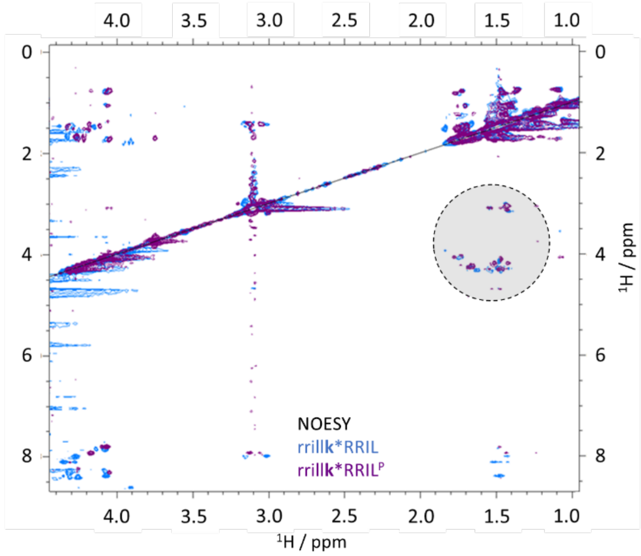

Figure S37. NOESY spectra of peptides rrillK*RRIL **4** (blue) and rrillk*RRIL **7** (purple). The compaction due to hydrophobic side-chain contacts is evidenced by the higher number of (blue) cross-peaks between the methyl and methylene moieties of **4** compared to **7**.

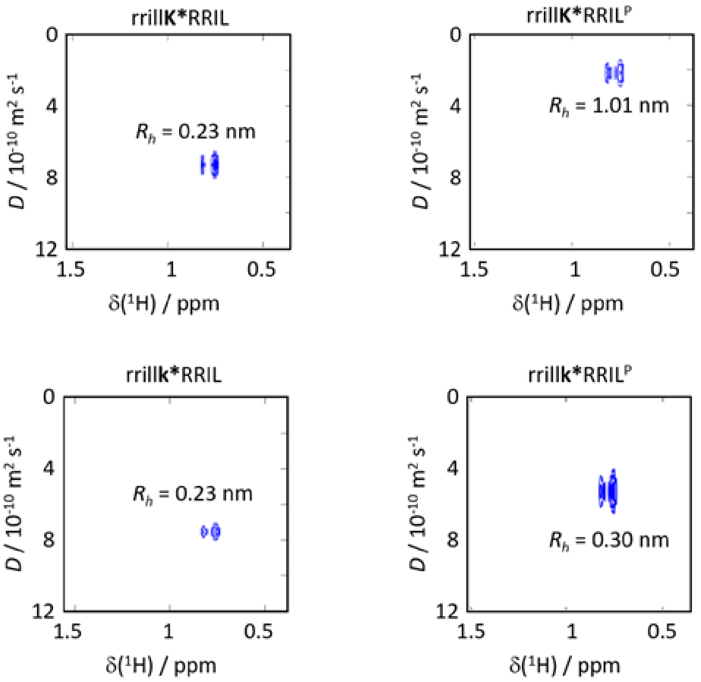

Figure S38. Diffusion coefficients and hydrodynamic radii found by DOSY for rrillK*RRIL **4** and rrillk*RRIL **7** in neat water and a 50 mM PBS.

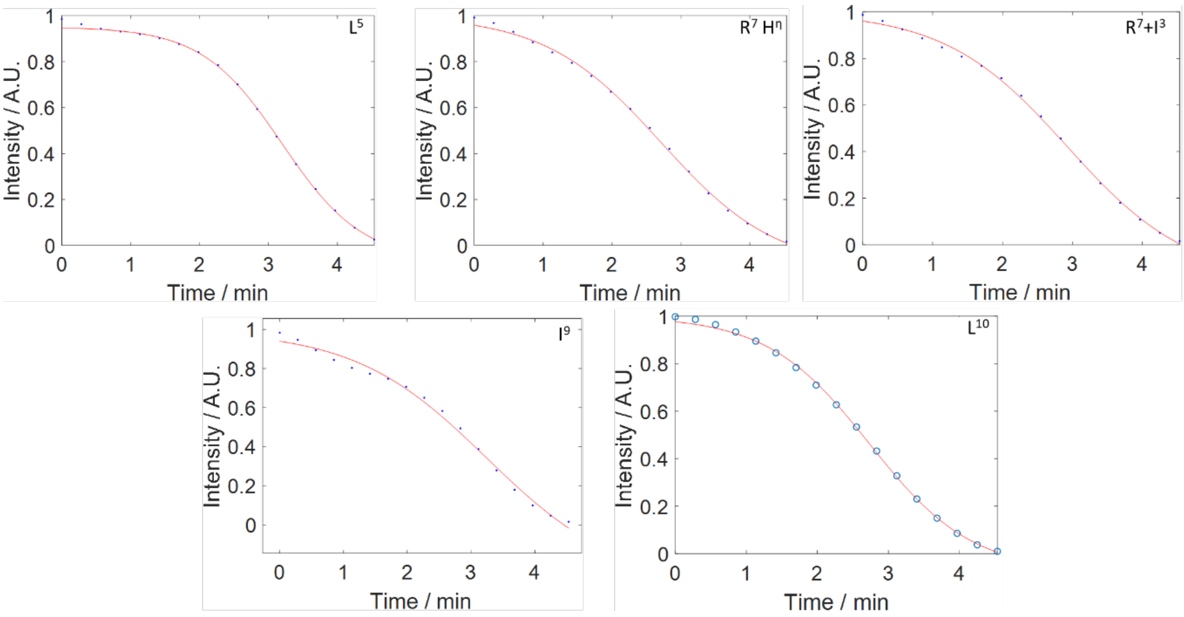

Figure S39. Sigmodial fits of peak real-time silica precipitation assay with the following equation $y = \frac{a}{1+10^{(b-t)*\sigma}} + d$,used to yield the cooperativity parameter σ for each peak visible for seen in Figure 6 of the main text.

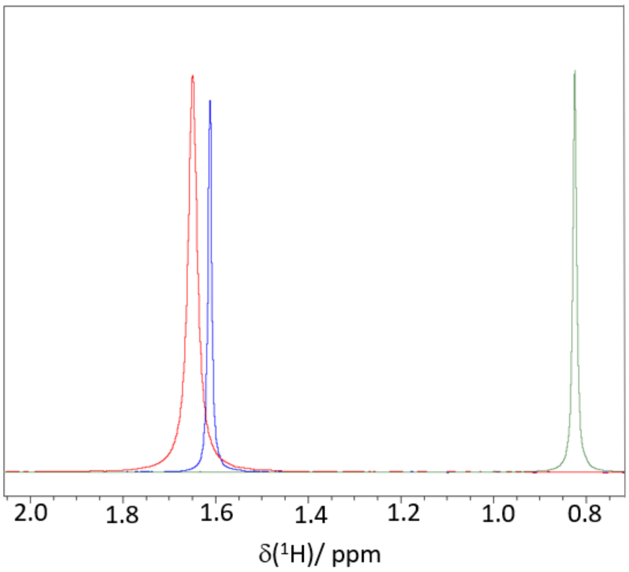

Figure S40. ^{31}P NMR phosphate signals observed in neat buffer (green) and in the presence of peptides **4** (blue) and **7** (red). The chemical shift changes upon peptide exposure indicating a direct interaction between the phosphate ions and the peptides.

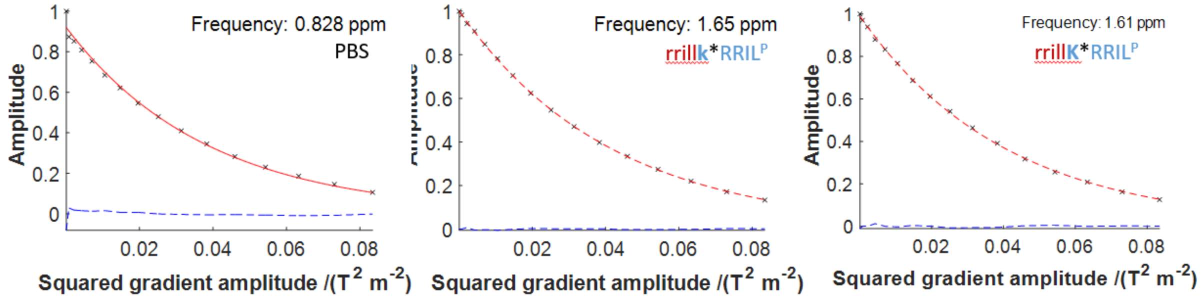

Figure S41. ^{31}P DOSY decay functions for neat buffer, (left), peptide **4** (center), and peptide **7** (right). The diffusion coefficients were fitted to 8.50±0.27, 7.84±0.03 and 8.04±0.05 nm^2/s. The presence of the peptides reduces the average diffusion coefficient indicating direct interaction between the peptide and the phosphate counterions.

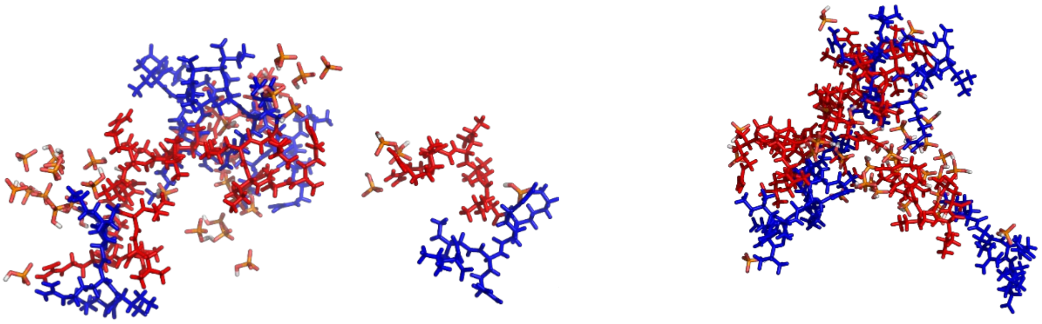

Figure S42. Structure after 700 ns from two MD replica of peptide **7**. Again, extended structures were observed structures compared to **4**. P_i ions again coordinate to the arginine residues.

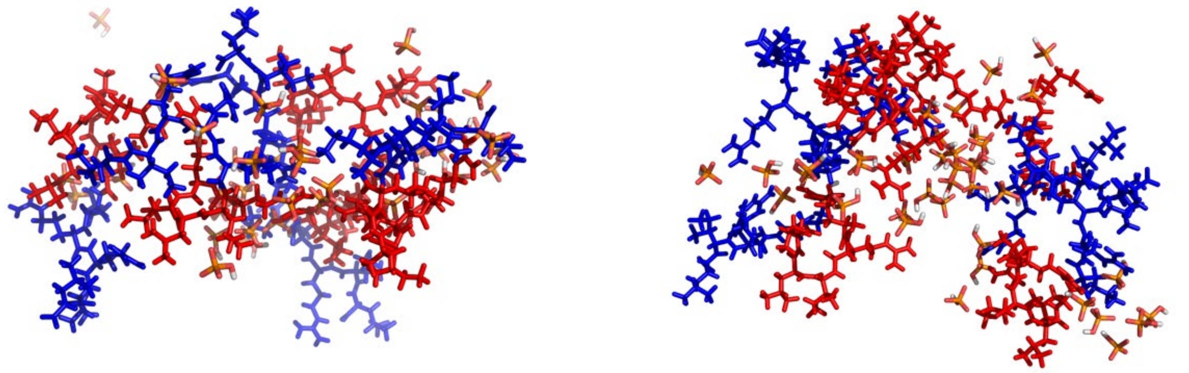

Figure S43. Structure after 700 ns from two MD replica of peptide **4**. Again, more globular structures compared to **7** were observed.

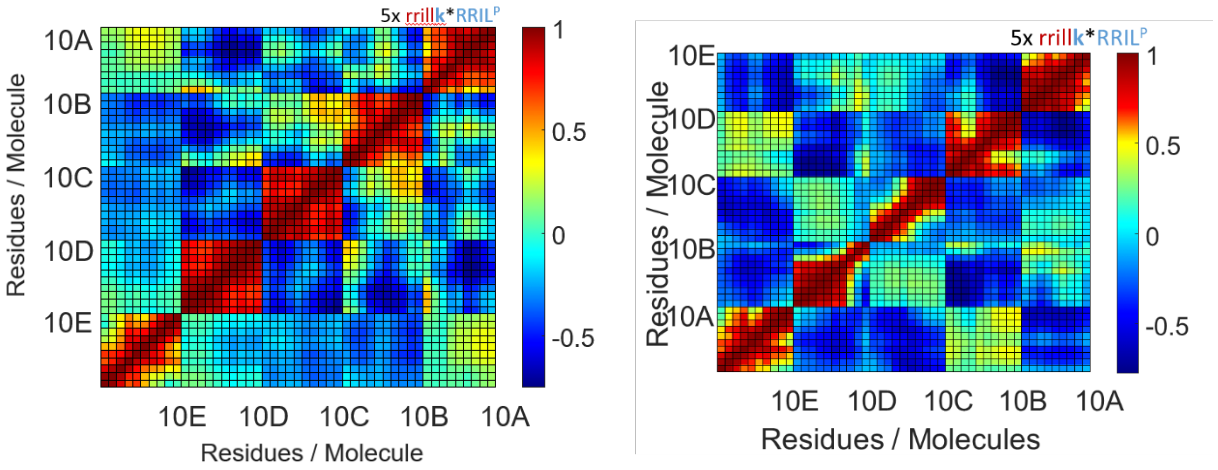

Figure S44. DCCM from two MD replica of peptide **4**. A high DCC between two residues indicates correlated motion and structural proximity.

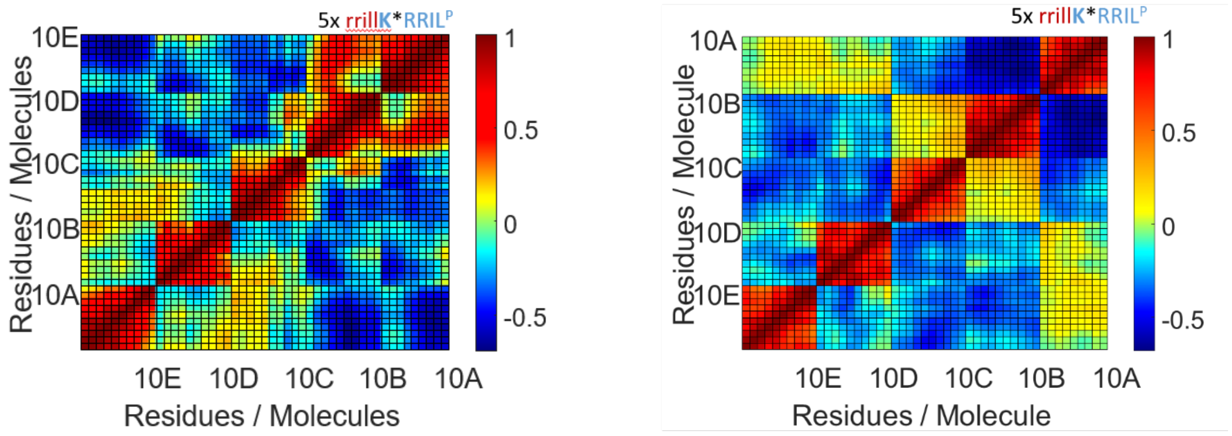

Figure S45. DCCM from two MD replica of peptide **7**. A high DCC between two residues indicates correlated motion and structural proximity.

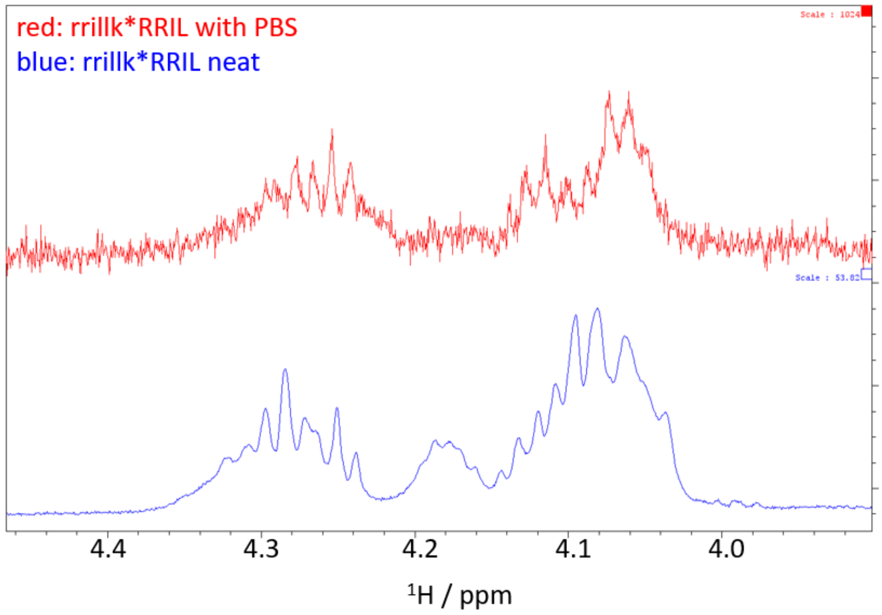

Figure S46. Alpha Proton chemical shifts for peptide **4,** in water (blue) and PBS (red). The chemical shift difference is below 0.02 ppm, indicating no significant changes in secondary structure propensities. Typical secondary chemical shifts upon β-sheet formation are on the order of 1 ppm. [3]

Supplementary References

[1] Nygaard M, Kragelund BB, Papaleo E, Lindorff-Larsen K. An Efficient Method for Estimating the Hydrodynamic Radius of Disordered Protein Conformations. Biophys J 2017;113:550–7. https://doi.org/10.1016/j.bpj.2017.06.042.

[2] Krieger E, Vriend G. YASARA View - molecular graphics for all devices - from smartphones to workstations. Bioinformatics 2014;30:2981–2. https://doi.org/10.1093/bioinformatics/btu426.

[3] Rule G, Hitchens K. Fundamentals of Protein NMR Spectroscopy. 1st ed. Springer, Dordrecht; 2006.
